# Light‐Activatable Ubiquitin for Studying Linkage‐Specific Ubiquitin Chain Formation Kinetics

**DOI:** 10.1002/advs.202406570

**Published:** 2024-12-24

**Authors:** Sudakshina Banerjee, Zeyneb Vildan Cakil, Kai Gallant, Johannes van den Boom, Shubhendu Palei, Hemmo Meyer, Malte Gersch, Daniel Summerer

**Affiliations:** ^1^ Department of Chemistry and Chemical Biology TU Dortmund University Otto‐Hahn Str. 4a 44227 Dortmund Germany; ^2^ Max Planck Institute of Molecular Physiology Chemical Genomics Center Otto‐Hahn Str. 15 44227 Dortmund Germany; ^3^ Center of Medical Biotechnology Faculty of Biology University of Duisburg‐Essen Universitätsstr. 2 45141 Essen Germany

**Keywords:** genetic code expansion, optochemical biology, small molecule inhibitors, ubiquitin

## Abstract

Ubiquitination is a dynamic post‐translational modification governing protein abundance, function, and localization in eukaryotes. The Ubiquitin protein is conjugated to lysine residues of target proteins, but can also repeatedly be ubiquitinated itself, giving rise to a complex code of ubiquitin chains with different linkage types. To enable studying the cellular dynamics of linkage‐specific ubiquitination, light‐activatable polyubiquitin chain formation is reported here. By incorporating a photocaged lysine at specific sites within ubiquitin through amber codon suppression, light‐dependent activation of ubiquitin chain extension is enabled for the monitoring of linkage‐specific polyubiquitination. The studies reveal rapid, minute‐scale ubiquitination kinetics for K11, K48, and K63 linkages. The role of individual components of the ubiquitin‐proteasome system in K48‐initiated chain synthesis is further studied by small molecule inhibition. The approach expands current perturbation strategies with the ability to control linkage‐specific ubiquitination with high temporal resolution and should find broad application for studying ubiquitinome dynamics.

## Graphical abstract

Ubiquitin dynamically forms differently linked polyubiquitin chains. To study rapid cellular kinetics of global linkage‐specific ubiquitination, light‐activatable polyubiquitin chain formation is reported via incorporation of a photocaged lysine. Minute‐scale ubiquitination kinetics for different linkages are observed, and the impact of ubiquitination factors on K48‐initiated chain formation is visualized.

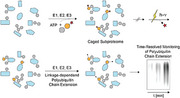

## 1 Introduction

Ubiquitination is a dynamic post‐translational modification in eukaryotes that acts as a versatile regulator of intracellular protein abundance, function, and localization.^[^ ^1^ ^]^ Ubiquitination involves conjugation of the small protein Ubiquitin (Ub) to target proteins via an isopeptide bond that connects the carboxyl group of the Ub C‐terminal glycine with the N^e^‐amino group of lysine residues on target proteins. However, conjugation can also occur at single or multiple lysines of Ub itself, giving rise to complex, linear or branched polyubiquitin chain topologies. This process is orchestrated by an enzymatic cascade involving activating E1 enzyme as well as different conjugating and ligating enzymes E2 and E3 that can exhibit specificity for single or multiple Ub lysine sites, resulting in chain topologies with distinct linkages. Moreover, Ub can be reversed by deubiquitinases (DUBs) with individual linkage specificities.^[^ ^2^ ^]^ Importantly, different Ub topologies on a target protein can result in distinct regulatory outputs, e.g. different signal strengths for proteasomal degradation,^[^ ^3^ ^]^ contributing to a complex and highly dynamic homeostasis.^[^ ^4^ ^]^

A deeper understanding of ubiquitination kinetics on a proteome‐wide level depends on effective perturbation methods that enable controlling and tracking linkage‐specific ubiquitination events with high temporal resolution. A number of strategies have greatly contributed to this area, such as pulse‐chase methods using, e.g., isotope‐labeled amino acids^[^ ^5^ ^]^ or fluorophores,^[^ ^6^ ^]^ capping with lysine‐less Ub,^[^ ^7^ ^]^ advances in mass spectrometry,^[^ ^8^ ^]^ chemically triggered ubiquitination of individual sites,^[^ ^9^ ^]^ and the use of small molecule binders and inhibitors of different components of the ubiquitin‐proteasome‐system (UPS).^[^ ^10^ ^]^ Such strategies can deliver invaluable insights into ubiquitinome dynamics at timescales defined by the rate of the respective perturbation or assay event, i.e., the expression rate of labeled proteins or diffusion and association rates of small molecules. It is however currently not possible to activate specific Ub lysine residues with light, which would allow the investigation of rapid polyubiquitin chain formation dynamics initiated by specific linkage types.

**Figure 1:**
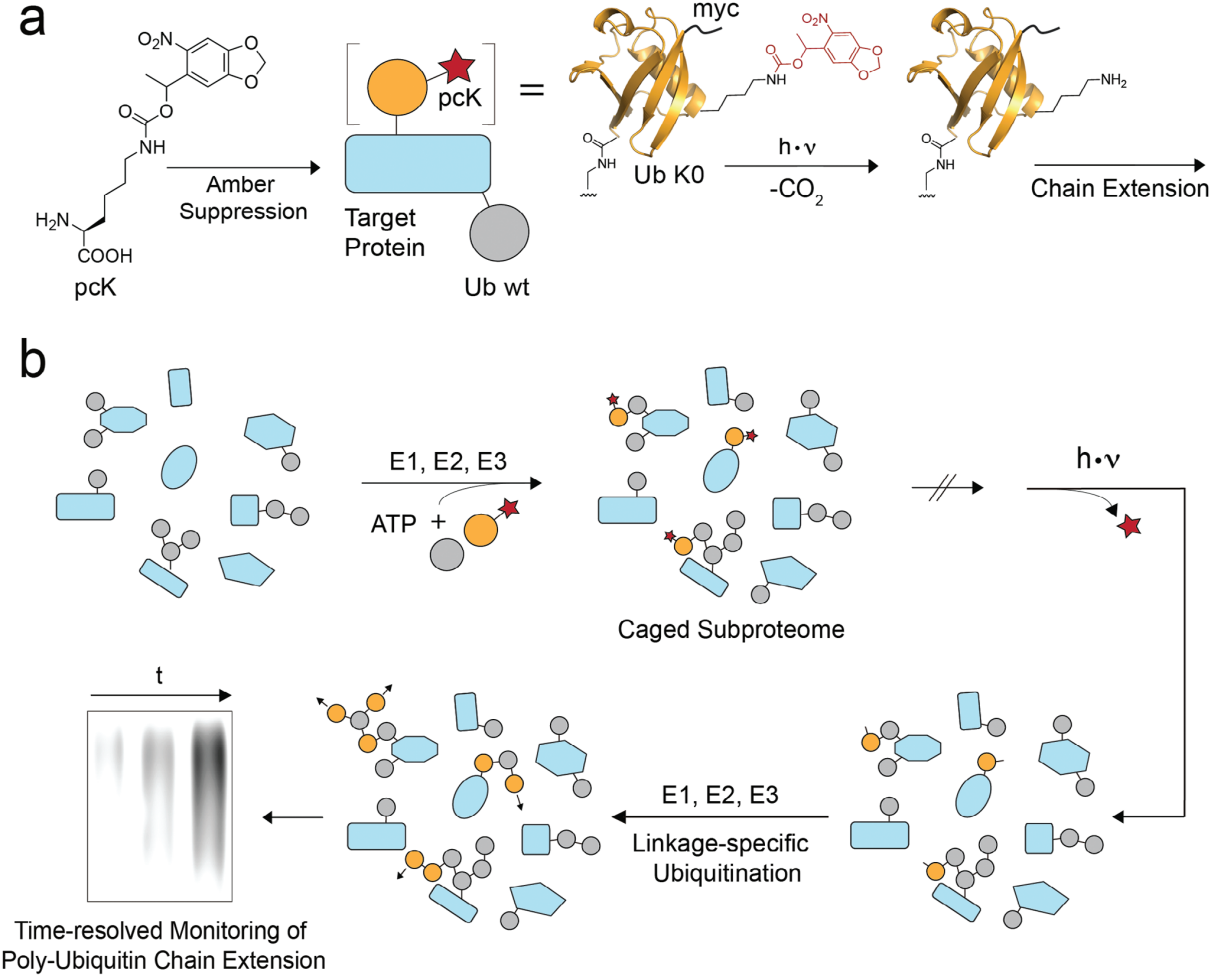
Light‐activated ubiquitination using a genetically encoded, photocaged lysine. a) Incorporation of photocaged lysine (pcK) into ubiquitin via amber suppression, its charging to target proteins, and its subsequent light‐decaging leads to polyubiquitination initiated by specific linkages. Ub K0: Ub with K→R mutations in all lysine sites except for the pcK site. b) Biosynthesis and light‐activation of a caged Ub subproteome that acts within the ubiquitin‐proteasome system (UPS). Inhibiting individual UPS components by small molecule inhibitors enables studying their function in early phases of *de novo* (poly)ubiquitination from specific linkages by rapid activation and time‐resolved monitoring.

To address this shortcoming, we here report light activation of linkage‐specific ubiquitin chain formation in mammalian cells.^[^ ^11^ ^]^ By expressing Ub variants bearing a single genetically encoded^[^ ^12^ ^]^ photocaged^[^ ^13^ ^]^ lysine (pcK, **Figure** **1**a) at specific sites, we generate a proteome subpopulation that is transiently caged for further ubiquitination at this site (Figure 1b). Removal of the photocaging group by a light pulse enables monitoring proteome‐wide (poly)ubiquitination initiated by a specific Ub linkage type (Figure 1c). We observe rapid ubiquitination kinetics on the minutes scale for K11, K48, and K63 linkages. Perturbation studies using small molecule inhibitors of different UPS components reveal their impact on early steps of K48‐initiated ubiquitinome synthesis.

## 2 Results and Discussion

**Figure 2:**
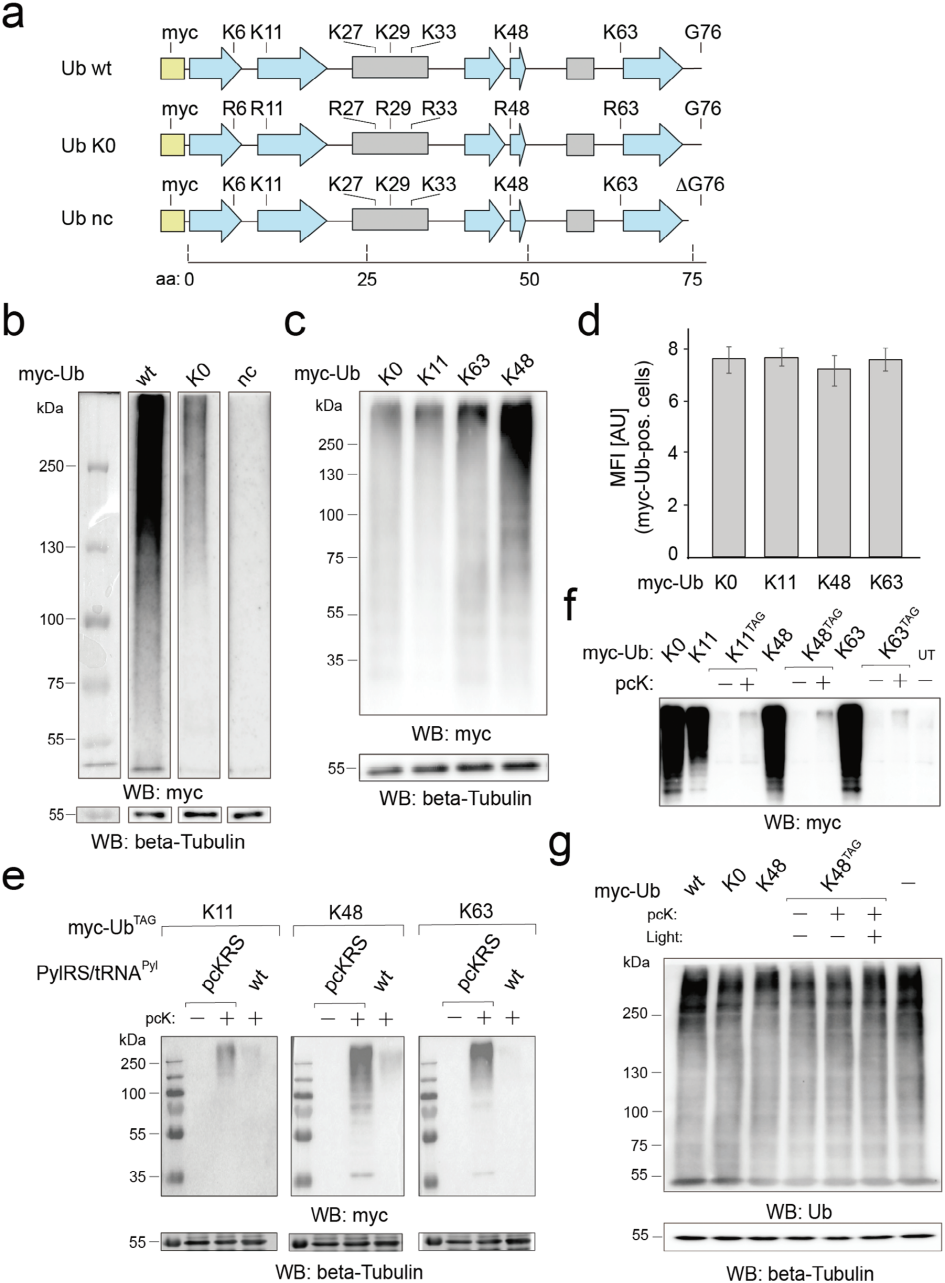
Analysis of caged Ub proteome subpopulation for light‐activation of linkage‐specific downstream ubiquitination a) Domain structure of Ub constructs used in this study. Blue arrows: b‐strands, grey boxes: a‐helices. b) SDS PAGE/anti‐myc blots of proteomes from HEK293T cells expressing indicated Ub variants for 24 h (all lanes are from the same blot with identical exposure time). Cells were treated with 10 µm MG132 5 h before harvesting (also applies to panels (c–g)). c) Experiment as in Figure 2b with indicated Ub linkage variants. d) Anti‐myc immunostaining and FACS analysis of cells from Figure 2c reveal similar total expression levels of Ub variants. MFI: Mean fluorescence intensity; AU: arbitrary units. e) High fidelity incorporation of pcK at three different Ub lysine sites in HEK293T cells expressing the indicated amber myc‐Ub variants along with the indicated pyrrolysyl‐tRNA‐synthetase pair for 24 h (individual contrast settings for each linkage). f) SDS PAGE/anti‐myc blots of proteomes from HEK293T cells expressing myc‐Ub variants with or without amber codon and in presence or absence of pcK for 24 h reveal minimal expression levels of caged Ub variants. g) SDS PAGE/anti‐Ub blots of proteomes from HEK293T cells expressing Ub variants for 24 h with or without amber codon and in presence or absence of pcK reveal minimal expression levels of Ub variants compared to the endogenous Ub pool. Cells expressing non amber Ub variants were harvested directly after 24 h and those expressing amber Ub variants were further treated with light and harvested 24 h later.

To establish our methodology, we focused on Ub variants bearing a single pcK residue at one of the three lysines K11, K48, and K63 because these represent the main ubiquitination sites most abundantly found in cellular Ub chain topologies with diverse and important functions.^[^ ^14^ ^]^ We introduced pcK into the variant Ub K0 (featuring K→R substitutions at all other K sites), which excludes ubiquitination of any other lysine sites. Although single‐lysine Ub restricts chain elongation at the mutated sites and thus alters chain topologies in the modified proteome subpopulation, it represents a functional Ub substrate compatible with further modification that is widely used (Figure 1).^[^ ^15^ ^]^ We expressed the variants with N‐terminal myc tags at low levels to obtain a minimal, selectively trackable Ub subpopulation that operates within the UPS. As an initial test, we individually expressed wild type Ub (Ub wt), Ub K0, and a Ub wt version that cannot be conjugated to lysines because of an altered C‐terminus (Ub nc, **Figure** **2**a; Figure S1, Supporting Information) in HEK293T cells, and conducted SDS PAGE analyses of the isolated whole proteomes. Anti‐myc blots revealed the proteome subpopulation modified with our Ub variants as a smear with a broad range of molecular weights (Figure 2b). As expected, the proteome fraction modified with Ub K0 thereby showed only a fraction (14%) of the intensity observed for Ub wt. No smear was observed for Ub nc, showing that the observed signal exclusively resulted from protein conjugates of the Ub variants (Figure 2b).

We next expressed Ub K11, K48 and K63 that each differ from K0 only in the single indicated R→K mutation, and thus allow further ubiquitination only in a linkage‐specific manner. All three variants resulted in similar or higher myc‐proteome intensities than Ub K0. K48 showed the highest levels, being in agreement with the generally high abundance of this linkage type in Ub topologies^[^ ^14^ ^]^ (Figure 2c). Anti‐myc immunostains and FACS analyses of the cells thereby revealed similar total expression levels for all four Ub variants, a scenario that allows attributing differences in the formed myc‐Ub proteome amounts to specific linkage preferences of the UPS rather than different availabilities of the Ub variants (Figure 2d; Figure S2, Supporting Information).

To incorporate pcK at Ub positions K11, K48, and K63, we co‐transfected vectors encoding the *Methanosarcina mazei* pyrrolysyl‐tRNA‐synthetase pair pcKRS/tRNA^Pyl^ that has been engineered to use pcK as substrate, with the respective Ub vectors containing a single in‐frame amber codon (TAG) at the incorporation target sites into HEK293T cells. We observed myc‐positive proteomes for all three amber positions when cells were cultivated in the presence of 0.32 mm pcK, but not in its absence, indicating faithful incorporation of pcK at all three target sites (Figure 2e). Moreover, we observed only a trace amount of myc‐positive Ub proteome when we replaced pcKRS with wt PylRS, for which pcK is a poor substrate (Figure 2e). Incorporation of pcK thereby resulted in a considerable further reduction of the Ub variant expression levels (compared to the non‐amber Ub variants, Figure 2f), and anti‐Ub blots did not differ between cells expressing pcK‐modified myc‐Ub or even non‐amber Ub (Figure 2g). These data collectively confirm that the caged myc‐Ub proteome indeed represents a minimal population with little potential to perturb the cellular UPS by extensive overexpression. Another potential source of proteome perturbation in genetic code expansion‐based cellular experiments is off‐target amber suppression leading to the formation of extended/misfolded endogenous proteins, but only a relatively small number of genes is found to actually be suppressed in related tRNA^Pyl^‐based systems.^[^ ^16^ ^]^

To characterize the Myc‐Ub‐containing pool of ubiquitin modifications further, we employed ubiquitin enrichment through the OtUBD reagent^[^ ^17^ ^]^ coupled to UbiCRest assays.^[^ ^18^ ^]^ We confirmed that the high molecular weight signal disappeared when eluates were incubated with the highly active deubiquitinase USP2. Incubation with the K48‐specific DUB OTUB1^*^ and with the K63‐specific DUB AMSH^*^ confirmed that Myc‐Ub was primarily added in polyubiquitin chains containing the two most abundant chain types (Figure S3, Supporting Information).

Of note, Ub proteins with caged lysines are able to be conjugated to substrates as monoubiquitination as well as be added to ubiquitin chains as distal tips, but prevent chain extensions in the absence of light. This priming of the system thus allows the specific assaying of the global linkage‐specific chain extension activity upon lysine decaging.

**Figure 3:**
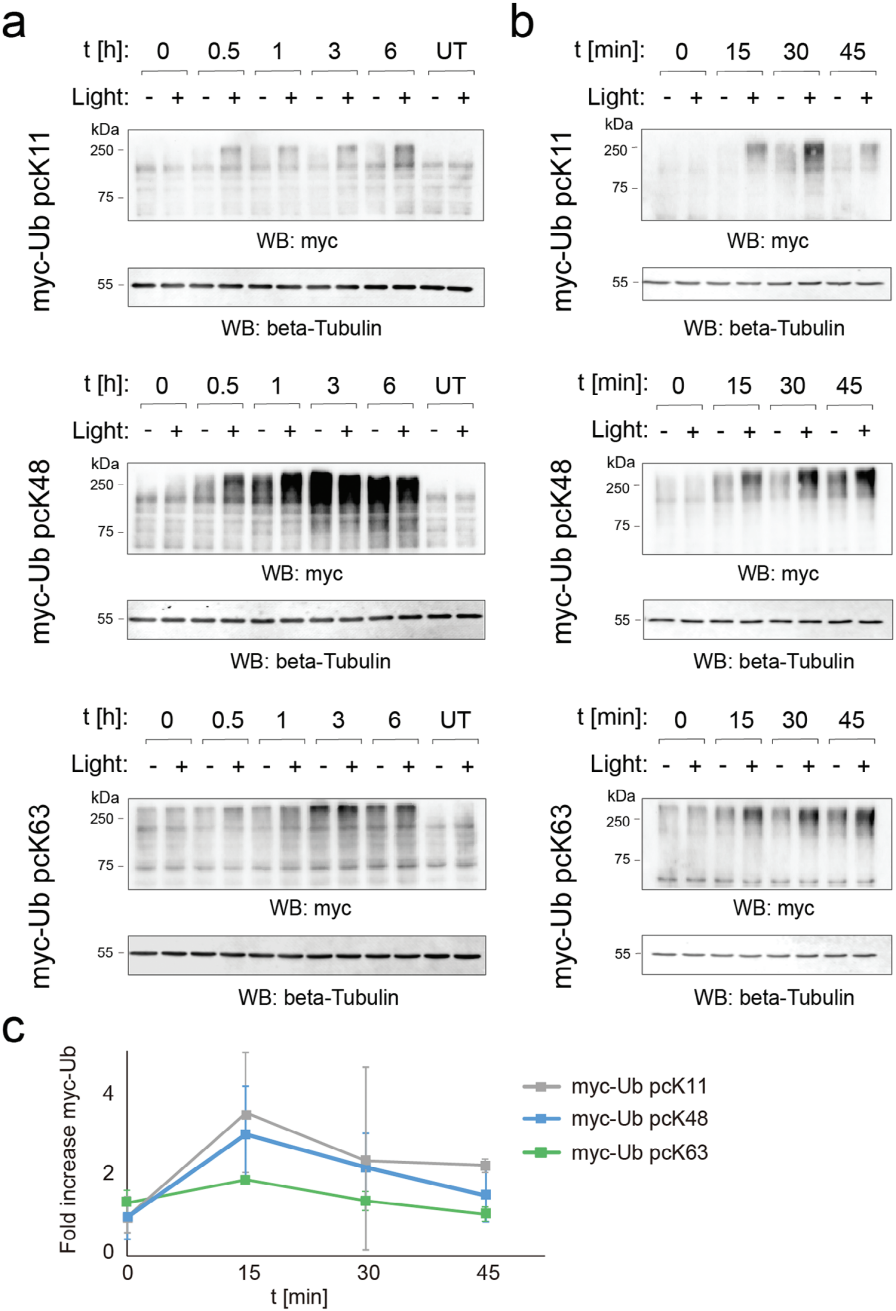
Light activation reveals rapid, proteome‐wide ubiquitination kinetics initiated by K11, K48, and K63 linkages. a) Long‐term, linkage‐specific ubiquitination kinetics after light activation of caged myc‐Ub variants (t = time after light). HEK293T cells were cultivated in the presence of 0.32 mm pcK for 24 h after transfection. After light activation (as stated in text), cells were grown in full media containing 25 µm MG132, lacking pcK, and harvested at each corresponding time‐point after light (also applies to panels (b,c)). b) Rapid, initial ubiquitination kinetics (<45 min after light). c) Plots of rapid, initial ubiquitination kinetics (myc‐Ub proteome intensities normalized to beta‐tubulin blot and no light control of each lane; error bars from N ≥ 2).

We next conducted light activation experiments with the Ub variants for studying linkage‐specific *de novo* ubiquitination kinetics over a window of 6 h. We transfected cells as above, cultivated them for 24 h in the presence of 0.32 mm pcK, exchanged the medium with warm DPBS lacking pcK to terminate expression of photocaged Ub, and irradiated the cells with light (365 nm, 4 min; see Figure S4, Supporting Information for irradiation kinetic reference experiment). We cultivated the cells further in full media containing 25 µm MG132 and lacking pcK, and harvested at each corresponding time‐point after light. We extracted proteomes and analyzed the formation of myc‐Ub proteomes by SDS‐PAGE/anti‐myc blots. To enable studying ubiquitinome synthesis kinetics uncoupled from overlaying proteasomal degradation, we conducted experiments in the presence of the proteasomal inhibitor MG132.^[^ ^19^ ^]^ We generally observed *de novo* myc‐ubiquitination in both irradiated and nonirradiated cells, with the latter however showing markedly slower increases. This indicates that a sufficiently high fraction of the proteome ubiquitination sites were modified with the photocaged Ub proteins, enabling selective monitoring of linkage‐specific *de novo* ubiquitination after light (**Figure** **3**a; Figure S5, Supporting Information). Importantly, cells grown under analogous conditions, but expressing non‐amber Ub K48 or K0 did not show any changes in the myc signals, confirming that the observed light‐dependent signal increases of high molecular weight myc‐Ub proteome were not an unspecific light‐response of the cells (Figure S6, Supporting Information). Moreover, we did not observe any effects of light on cell morphology or viability (Figures S7–S8, Supporting Information).

Overall, the observed kinetics were rapid for all three linkage types, with large increases already after 0.5 h. The three linkage types however strongly differed in the amount of formed myc‐Ub proteome, with K48 again showing the highest signals. This is in contrast with the similar ubiquitination levels observed for the three linkage types before light activation that can occur at any lysine of target proteins and pre‐existing Ub chains, and thus should involve diverse UPS enzymes (Figure 2f). The different myc‐Ub proteome amounts observed for the linkages after light‐activation thus likely reflect the differences in activity of the specific involved UPS enzymes for each linkage type. Of note, the myc‐Ub proteome amounts synthesized from available free lysine residues alone (‐ light) reached similar levels as the ones from both free and decaged lysines combined (+ light), presumably due to the concurrent proteasome inhibition that depletes monomeric Ub. This becomes visible by comparing −/+ light for pcK48 and pcK63, whereas for pcK11, no saturation is reached within the observation time window (Figure 3a).

We next conducted kinetic measurements with a short time window of up to 45 min post light, and observed myc‐Ub proteome increases already after 15 min, confirming a very rapid build‐up of Ub proteome from all three linkages (Figure 3b; Figure S9, Supporting Information). To reveal light‐induced, linkage‐specific synthesis kinetics independently of the observed background synthesis, we normalized the myc signals from irradiated cells to the nonirradiated cells of each time point. These kinetics – though differing between the linkages – generally revealed an initial burst with a maximum at 15 min in all cases, and a subsequent stabilization in relation to the background (Figure 3c).

**Figure 4:**
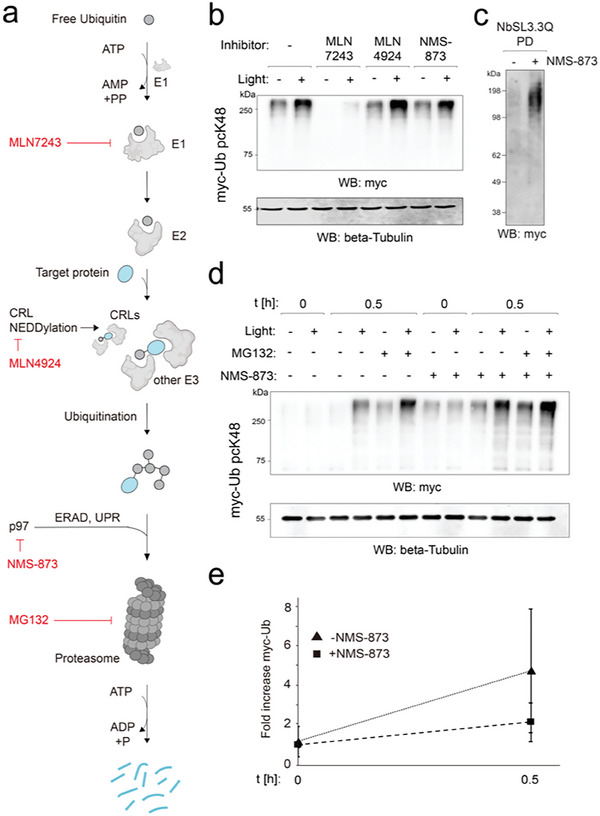
Ubiquitin light activation in combination with small molecule UPS inhibitors enables studying the involvement of individual UPS components on rapid, K48‐linked *de novo* polyubiquitination. a) Scheme of UPS synthesis/degradation pathway with highlighted UPS factors targeted by used inhibitors. b) Effects of UPS inhibitors on early, K48‐specific *de novo* ubiquitinome synthesis (t = 0.5 h) in presence of MG132. HEK293T cells were cultivated in the presence of 0.32 mm pcK for 24 h after transfection. The inhibitors were added to the media in concentrations as mentioned. Following the pretreatment times, light activation was performed and cells were grown in full media containing 25 µm MG132 and the respective inhibitors, lacking pcK, and harvested at t = 0.5 h after light (also applies to panel (e)). c) Enrichment of branched ubiquitin with agarose‐immobilized NbSL3.3Q nanobody from HEK293T cells with light‐decaged myc‐Ub pcK48, with or without prior addition of the p97 inhibitor NMS‐873 (5 µm for 0.5 h). Eluates were analyzed by western blotting for Myc. d,e) Effect of NMS873 on early, K48‐specific *de novo* ubiquitinome synthesis in presence and absence of MG132.

We next applied our methodology for the comparison of cellular polyubiquitin synthesis capacities in different cellular states. Small molecule inhibitors of UPS components hold great therapeutic potential and are central perturbation tools to study the cellular roles of the UPS.^[^ ^10a^ ^]^ The use of inhibitors in our approach offers the investigation of the involvement of individual UPS components in the observed rapid, linkage‐specific *de novo* synthesis of Ub chains. We employed three small molecules perturbing different steps of the ubiquitination/proteasomal degradation pathway: MLN7243^[^ ^20^ ^]^ as a mechanism based, AMP‐mimicking inhibitor of both mammalian E1 enzymes (UAE and UBA6),^[^ ^21^ ^]^ MLN4924^[^ ^22^ ^]^ as a covalent inhibitor of the NEDD8‐activating enzyme (NAE) to inhibit Cullin‐Ring‐E3 ligase activation, and NMS‐873 as an inhibitor of the AAA^+^ ATPase VCP/p97. We focused on measuring Ub chains initiated by the abundant K48 linkage under conditions of proteasomal inhibition by MG132 (**Figure** **4**a).

Use of MLN7243 allowed effective starving of cellular Ub pools and shutdown of downstream ubiquitination events (Figure 4a,b). Interestingly, a residual *de novo* ubiquitination was however still visible even after 6 h pre‐incubation with 1 µm MLN7243, indicating that low levels of active E1 and/or charged Ub are still available under these widely used treatment conditions (Figure 4b).

We next employed MLN4924,^[^ ^22^ ^]^ a covalent inhibitor of the NEDD8‐activating enzyme (NAE). NAE‐dependent NEDDylation controls Cullin‐RING‐E3 ligases (CRL), the largest E3 ligase class that regulates proteins with important functions in cancer cell growth and survival pathways (Figure 4a).^[^ ^23^ ^]^ Interestingly, we did not observe a change in global K48‐linked polyubiquitin kinetics even after a 4 h preincubation with 1 µm MLN4924 (Figure 4b). This suggests that a large part of the observed, K48‐initiated chain extension ubiquitination occurs independently of CRL activity,^[^ ^24^ ^]^ in agreement with previous observations.^[^ ^22^ ^]^ Finally, we studied NMS‐873,^[^ ^25^ ^]^ an inhibitor of the AAA^+^ ATPase VCP/p97 that promotes proteasomal degradation of ubiquitinated client proteins by their extraction from membranes or complexes, and possesses co‐factors with K48‐linked Ub chains specificity^[^ ^26^ ^]^ (Figure 4a). Treatment with 5 µm NMS‐873 for 4 h did not result in a reduced K48‐initiated ubiquitinome synthesis rate in the presence of MG132, being in agreement with the requirement of proteasome activity for client protein degradation (Figure 4b). Inhibition of p97 was recently reported to cause the accumulation of K48‐K63‐branched polyubiquitin.^[^ ^27^ ^]^ We, therefore, assessed the compatibility of our method with the analysis of branched ubiquitin by employing a nanobody specific for K48‐K63‐branched ubiquitin,^[^ ^27^ ^]^ demonstrating the emergence of Myc‐Ub signal in pulldown fractions (Figure 4c). In addition, experiments in the absence of MG132 afforded a marked increase of myc‐Ub proteome levels in the presence of NMS‐873, directly revealing the action of VCP/p97 as counterpart of K48‐linked *de novo* ubiquitinome synthesis on a rapid timescale (Figure 4d,e; Figure S10, Supporting Information).

## 3 Conclusion

We here report light activation of ubiquitin chain extension in mammalian cells. Incorporation of a genetically encoded photocaged lysine via an engineered PylRS/tRNA^Pyl^ pair enabled the expression of tagged Ub variants with individual, transiently inactive ubiquitination sites that are employed by the UPS to synthesize a small, trackable sub‐ubiquitinome.

Light activation triggers ubiquitination from the decaged lysine sites, enabling monitoring of proteome *de novo* ubiquitination initiated by specific linkages. Light activation studies revealed the synthesis of distinct ubiquitinome levels for K11, K48, and K63 linkages with rapid, burst‐like kinetics that result in relative maxima already after 15 min for all linkages. Inhibition of individual UPS components responsible for the synthesis of activated Ub and CRL activation (E1 and NAE, respectively), or for the unfolding of K48‐ubiquitinated client proteins for proteasome‐dependent degradation by (VCP/p97) revealed the impact of these components on K48‐initiated ubiquitinome synthesis on the low minutes time scale. The high temporal resolution of our approach and its ability to selectively control individual linkage‐types adds new abilities to the current toolset of perturbation strategies, and is compatible with diverse downstream analysis techniques to study rapid, linkage specific ubiquitination dynamics for individual proteins as well as on global proteomic levels. Given the role of Ub in regulating protein homeostasis, function and localization in a vast number of normal and disease‐related processes, we anticipate that our approach will find wide use for studying the dynamics of linkage‐specific ubiquitination.

## Conflict of Interest

The authors declare no conflict of interest.

## Supporting information



Supporting Information

## Data Availability

The data that support the findings of this study are available from the corresponding author upon reasonable request.

## References

[1] C. Pohl , I. Dikic , Science 2019, 366, 818. 31727826 10.1126/science.aax3769

[2] M. J. Clague , S. Urbé , D. Komander , Nat. Rev. Mol. Cell Biol. 2019, 20, 338. 30733604 10.1038/s41580-019-0099-1

[3] A. Ciechanover , A. Stanhill , Bba Mol. Cell Res. 2014, 1843, 86. 10.1016/j.bbamcr.2013.07.007 23872423

[4] J. Lutz , E. Hollmuller , M. Scheffner , A. Marx , F. Stengel , Angew. Chem. Int. Ed. Engl. 2020, 59, 12371. 32301549 10.1002/anie.202003058 PMC7384046

[5] a) W. Kim , E. J. Bennett , E. L. Huttlin , A. Guo , J. Li , A. Possemato , M. E. Sowa , R. Rad , J. Rush , M. J. Comb , J. W. Harper , S. P. Gygi , Mol. Cell. 2011, 44, 325; 21906983 10.1016/j.molcel.2011.08.025 PMC3200427

[6] A. A. Kudriaeva , I. Livneh , M. S. Baranov , R. H. Ziganshin , A. E. Tupikin , S. O. Zaitseva , M. R. Kabilov , A. Ciechanover , A. A. Belogurov Jr , Cell. Chem. Biol. 2021, 28, 1192. 33675681 10.1016/j.chembiol.2021.02.009

[7] K. Oshikawa , M. Matsumoto , K. Oyamada , K. I. Nakayama , J. Proteome Res. 2012, 11, 796. 22053931 10.1021/pr200668y

[8] G. Prus , S. Satpathy , B. T. Weinert , T. Narita , C. Choudhary , Cell 2024, 187, 2875. 38626770 10.1016/j.cell.2024.03.024 PMC11136510

[9] M. Fottner , A. D. Brunner , V. Bittl , D. Horn‐Ghetko , A. Jussupow , V. R. I. Kaila , A. Bremm , K. Lang , Nat. Chem. Biol. 2019, 15, 276. 30770915 10.1038/s41589-019-0227-4

[10] a) D. H. Lee , A. L. Goldberg , Trends Cell Biol. 1998, 8, 397; 9789328 10.1016/s0962-8924(98)01346-4

[11] a) N. Ankenbruck , T. Courtney , Y. Naro , A. Deiters , Angew. Chem. Int. Ed. Engl. 2018, 57, 2768; 28521066 10.1002/anie.201700171 PMC6026863

[12] a) H. Xiao , P. G. Schultz , Cold Spring Harb. Perspect. Biol. 2016, 8, a023945; 27413101 10.1101/cshperspect.a023945 PMC5008073

[13] a) E. A. Lemke , D. Summerer , B. H. Geierstanger , S. M. Brittain , P. G. Schultz , Nat. Chem. Biol. 2007, 3, 769; 17965709 10.1038/nchembio.2007.44

[14] I. Dikic , B. A. Schulman , Nat. Rev. Mol. Cell Biol. 2023, 24, 273. 36284179 10.1038/s41580-022-00543-1 PMC9595094

[15] a) I. Ziv , Y. Matiuhin , D. S. Kirkpatrick , Z. Erpapazoglou , S. Leon , M. Pantazopoulou , W. Kim , S. P. Gygi , R. Haguenauer‐Tsapis , N. Reis , M. H. Glickman , O. Kleifeld , Mol. Cell. Proteomics 2011, 10, M111.009753. 10.1074/mcp.M111.009753 PMC3098606 21427232

[16] M. D. Bartoschek , E. Ugur , T. A. Nguyen , G. Rodschinka , M. Wierer , K. Lang , S. Bultmann , Nucleic Acids Res. 2021, 49, e62. 33684219 10.1093/nar/gkab132 PMC8216290

[17] M. Zhang , J. M. Berk , A. B. Mehrtash , J. Kanyo , M. Hochstrasser , PLoS Biol. 2022, 20, e3001501. 35771886 10.1371/journal.pbio.3001501 PMC9278747

[18] a) T. E. Mevissen , M. K. Hospenthal , P. P. Geurink , P. R. Elliott , M. Akutsu , N. Arnaudo , R. Ekkebus , Y. Kulathu , T. Wauer , F. El Oualid , S. M. Freund , H. Ovaa , D. Komander , Cell 2013, 154, 169; 23827681 10.1016/j.cell.2013.05.046 PMC3705208

[19] K. L. Rock , C. Gramm , L. Rothstein , K. Clark , R. Stein , L. Dick , D. Hwang , A. L. Goldberg , Cell 1994, 78, 761. 8087844 10.1016/s0092-8674(94)90462-6

[20] M. L. Hyer , M. A. Milhollen , J. Ciavarri , P. Fleming , T. Traore , D. Sappal , J. Huck , J. Shi , J. Gavin , J. Brownell , Y. Yang , B. Stringer , R. Griffin , F. Bruzzese , T. Soucy , J. Duffy , C. Rabino , J. Riceberg , K. Hoar , A. Lublinsky , S. Menon , M. Sintchak , N. Bump , S. M. Pulukuri , S. Langston , S. Tirrell , M. Kuranda , P. Veiby , J. Newcomb , P. Li , et al., Nat. Med. 2018, 24, 186. 29334375 10.1038/nm.4474

[21] J. Jin , X. Li , S. P. Gygi , J. W. Harper , Nature 2007, 447, 1135. 17597759 10.1038/nature05902

[22] T. A. Soucy , P. G. Smith , M. A. Milhollen , A. J. Berger , J. M. Gavin , S. Adhikari , J. E. Brownell , K. E. Burke , D. P. Cardin , S. Critchley , C. A. Cullis , A. Doucette , J. J. Garnsey , J. L. Gaulin , R. E. Gershman , A. R. Lublinsky , A. McDonald , H. Mizutani , U. Narayanan , E. J. Olhava , S. Peluso , M. Rezaei , M. D. Sintchak , T. Talreja , M. P. Thomas , T. Traore , S. Vyskocil , G. S. Weatherhead , J. Yu , J. Zhang , et al., Nature 2009, 458, 732. 19360080 10.1038/nature07884

[23] J. E. Brownell , M. D. Sintchak , J. M. Gavin , H. Liao , F. J. Bruzzese , N. J. Bump , T. A. Soucy , M. A. Milhollen , X. F. Yang , A. L. Burkhardt , J. Y. Ma , H. K. Loke , T. Lingaraj , D. Y. Wu , K. B. Hamman , J. J. Spelman , C. A. Cullis , S. P. Langston , S. Vyskocil , T. B. Sells , W. D. Mallender , I. Visiers , P. Li , C. F. Claiborne , M. Rolfe , J. B. Bolen , L. R. Dick , Mol. Cell 2010, 37, 102. 20129059 10.1016/j.molcel.2009.12.024

[24] E. J. Bennett , J. Rush , S. P. Gygi , J. W. Harper , Cell 2010, 143, 951. 21145461 10.1016/j.cell.2010.11.017 PMC3008586

[25] P. Magnaghi , R. D'Alessio , B. Valsasina , N. Avanzi , S. Rizzi , D. Asa , F. Gasparri , L. Cozzi , U. Cucchi , C. Orrenius , P. Polucci , D. Ballinari , C. Perrera , A. Leone , G. Cervi , E. Casale , Y. Xiao , C. Wong , D. J. Anderson , A. Galvani , D. Donati , T. O'Brien , P. K. Jackson , A. Isacchi , Nat. Chem. Biol. 2013, 9, 548. 23892893 10.1038/nchembio.1313

[26] M. Locke , J. I. Toth , M. D. Petroski , Biochem. J. 2014, 459, 205. 24417208 10.1042/BJ20120662 PMC4160081

[27] S. M. Lange , M. R. McFarland , F. Lamoliatte , T. Carroll , L. Krshnan , A. Perez‐Rafols , D. Kwasna , L. Shen , I. Wallace , I. Cole , L. A. Armstrong , A. Knebel , C. Johnson , V. De Cesare , Y. Kulathu , Nat. Struct. Mol. Biol. 2024, 31, 1872. 38977901 10.1038/s41594-024-01354-y PMC11638074

