# Supplementary material for: Light‐Activatable Ubiquitin for Studying Linkage‐Specific Ubiquitin Chain Formation Kinetics
Source: Adv Sci (Weinh). 2024 Dec 24;12(6):2406570. doi: 10.1002/advs.202406570 (PMC11809417; doi:10.1002/advs.202406570)
Supplement: Supplementary file 1 — Supporting Information [file ADVS-12-2406570-s001.pdf]

ADVANCED SCIENCE
Open Access

Supporting Information

for *Adv. Sci.*, DOI 10.1002/advs.202406570

Light-Activatable Ubiquitin for Studying Linkage-Specific Ubiquitin Chain Formation Kinetics

Sudakshina Banerjee, Zeyneb Vildan Cakil, Kai Gallant, Johannes van den Boom, Shubhendu Palei, Hemmo Meyer, Malte Gersch and Daniel Summerer**

Light-Activatable Ubiquitin for Studying Linkage-Specific Ubiquitin Chain Formation Kinetics

Sudakshina Banerjee[a], Zeyneb Vildan Cakil[a], Kai Gallant[a,b], Johannes van den Boom[c], Shubhendu Palei[a], Hemmo Meyer[c], Malte Gersch*[a,b], and Daniel Summerer*[a]

[a] Sudakshina Banerjee, Kai Gallant, Dr. Malte Gersch, Prof. Dr. Daniel Summerer
Department of Chemistry and Chemical Biology, TU Dortmund University
Otto-Hahn Str. 4a, 44227 Dortmund, Germany
E-mail: malte.gersch@tu-dortmund.de and daniel.summerer@tu-dortmund.de

[b] Kai Gallant, Dr. Malte Gersch
Max Planck Institute of Molecular Physiology, Chemical Genomics Center
Otto-Hahn Str. 15, 44227 Dortmund, Germany

[c] Dr. Johannes van den Boom, Prof. Dr. Hemmo Meyer
Center of Medical Biotechnology, Faculty of Biology, University of Duisburg-Essen
Universitätsstr. 2, 45141 Essen, Germany

Supplementary Information

TABLE OF CONTENTS

SUPPLEMENTARY FIGURES 3

FIGURE S1. PLASMID MAPS OF EXPRESSION VECTORS USED IN THIS STUDY. 3

MAP OF THE EXPRESSION VECTOR FOR A) N-TERMINALLY MYC-TAGGED UB WT (P2945). THE MYC-TAGGED MUTANTS OF UB, INCLUDING UB K0, UB K11, UB K48, UB K63, UB K11^{TAG}, UB K48^{TAG}, AND UB K63^{TAG}, SHARE THE SAME VECTOR FEATURES EXCEPT FOR THE INDICATED MUTATIONS, B) MODIFIED PCKRS (P2946). 3

FIGURE S2. REPRESENTATIVE FACS DENSITY PLOTS AND ALTERNATE ANALYSES SHOWING THE SIMILAR EXPRESSION LEVELS OF MYC UB K0 AND OTHER SINGLE LYSINE UB VARIANTS IN HEK293T CELLS. 4

FIGURE S3. ENRICHMENT AND UBIQUITIN LINKAGE ANALYSIS OF MYC-UB-CONTAINING CELLULAR POLYUBIQUITIN. 5

FIGURE S4. IN VITRO DECAGING KINETICS OF PCK IN THE EXPERIMENTAL SETUP USED FOR CELLULAR DECAGING STUDIES. 6

FIGURE S5. ADDITIONAL SDS PAGE/ANTI-MYC BLOTS FOR THE ANALYSES OF LONG-TERM, LINKAGE-SPECIFIC UBIQUITINATION KINETICS AFTER LIGHT ACTIVATION OF CAGED UB VARIANTS. 7

FIGURE S6. LIGHT ALONE OR MG132 DO NOT HAVE AN EFFECT ON THE INCREASE IN HIGH MOLECULAR WEIGHT MYC-UB IN THE PROTEOME. 8

FIGURE S7. NO EFFECT OF LIGHT ON CELL MORPHOLOGY. 9

FIGURE S8. NO EFFECT OF LIGHT ON CELL VIABILITY 9

FIGURE S9. ADDITIONAL SDS PAGE/ANTI-MYC BLOTS FOR THE ANALYSES OF SHORT-TERM, LINKAGE-SPECIFIC UBIQUITINATION KINETICS AFTER LIGHT ACTIVATION OF CAGED UB VARIANTS. 10

FIGURE S10. ADDITIONAL SDS PAGE/ANTI-MYC BLOT FOR THE ANALYSES OF EFFECT OF NMS873 ON EARLY, K48-SPECIFIC *DE NOVO* UBIQUITOME SYNTHESIS (T=0.5 H) IN PRESENCE OF MG132. 11

FIGURE S11. CHANGES IN MONO MYC-UB POOL IN HEK293T CELLS EXPRESSING MYC-UB PCK48 IN PRESENCE OR ABSENCE OF PCK, WITH OR WITHOUT LIGHT IRRADIATION. 12

FIGURE S8. SOURCE DATA OF ALL IMMUNOBLOTS PERFORMED IN THIS WORK. 16

MATERIALS AND METHODS 17

SUPPLEMENTARY TABLES 21

SUPPLEMENTARY REFERENCES 23

SUPPLEMENTARY FIGURES

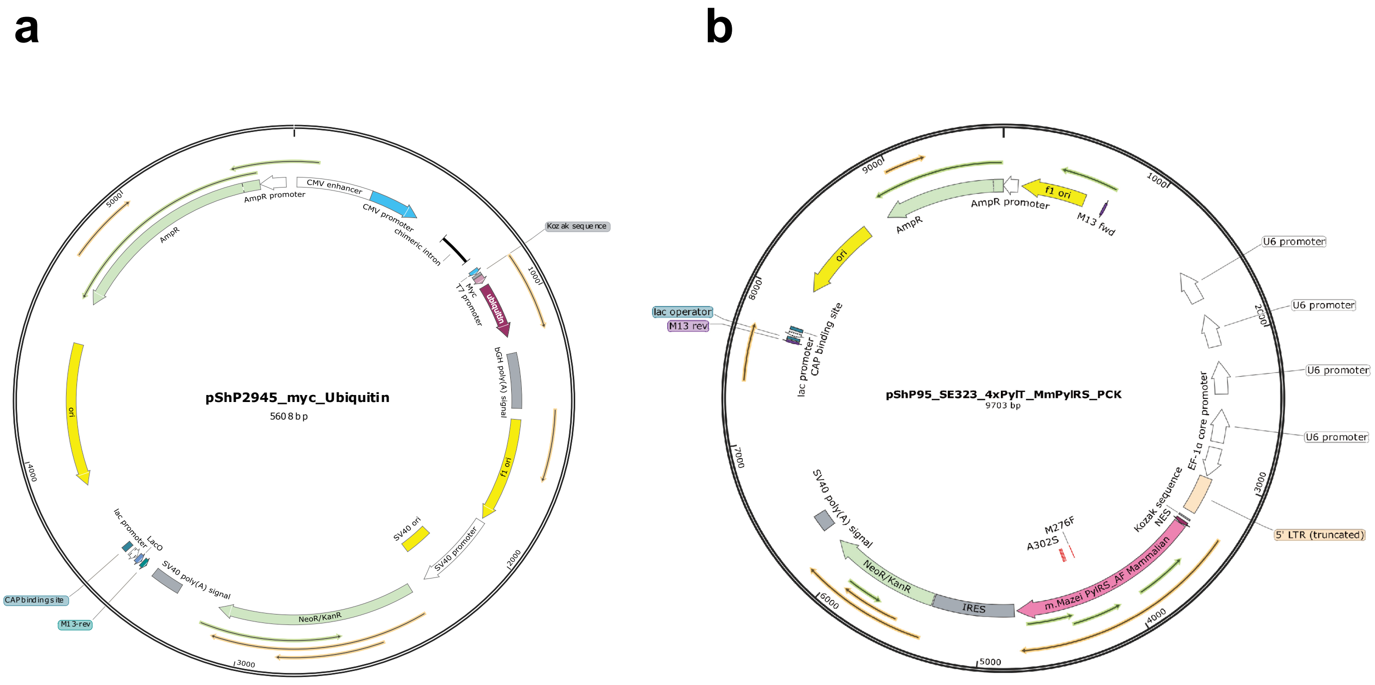

Figure S1. Plasmid maps of expression vectors used in this study.

Map of the expression vector for a) N-terminally myc-tagged Ub WT (p2945). The myc-tagged mutants of Ub, including Ub K0, Ub K11, Ub K48, Ub K63, Ub K11^{TAG}, Ub K48^{TAG}, and Ub K63^{TAG}, share the same vector features except for the indicated mutations, b) modified pcKRS (p2946).

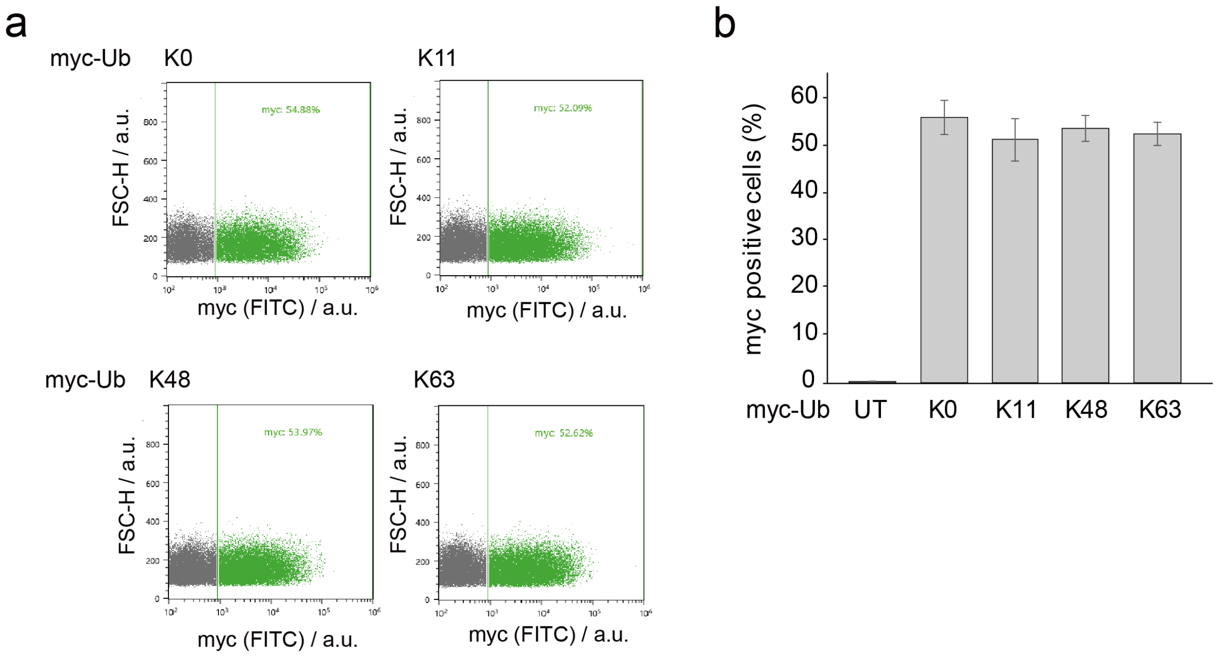

Figure S2. Representative FACS density plots and alternate analyses showing the similar expression levels of myc Ub K0 and other single lysine Ub variants in HEK293T cells.

a) The myc positive cell population of HEK293T cells expressing myc Ub K0, K11, K48 and K63 for 24 hours, were analyzed by FCM. Green lines indicate the intensity thresholds determined by the fluorescence intensity of untransfected cells. **b)** Bar diagrams showing the mean of the percentage of myc positive cells. Error bars from N=3 biological replicates. UT= untransfected cells. a.u. = arbitrary units.

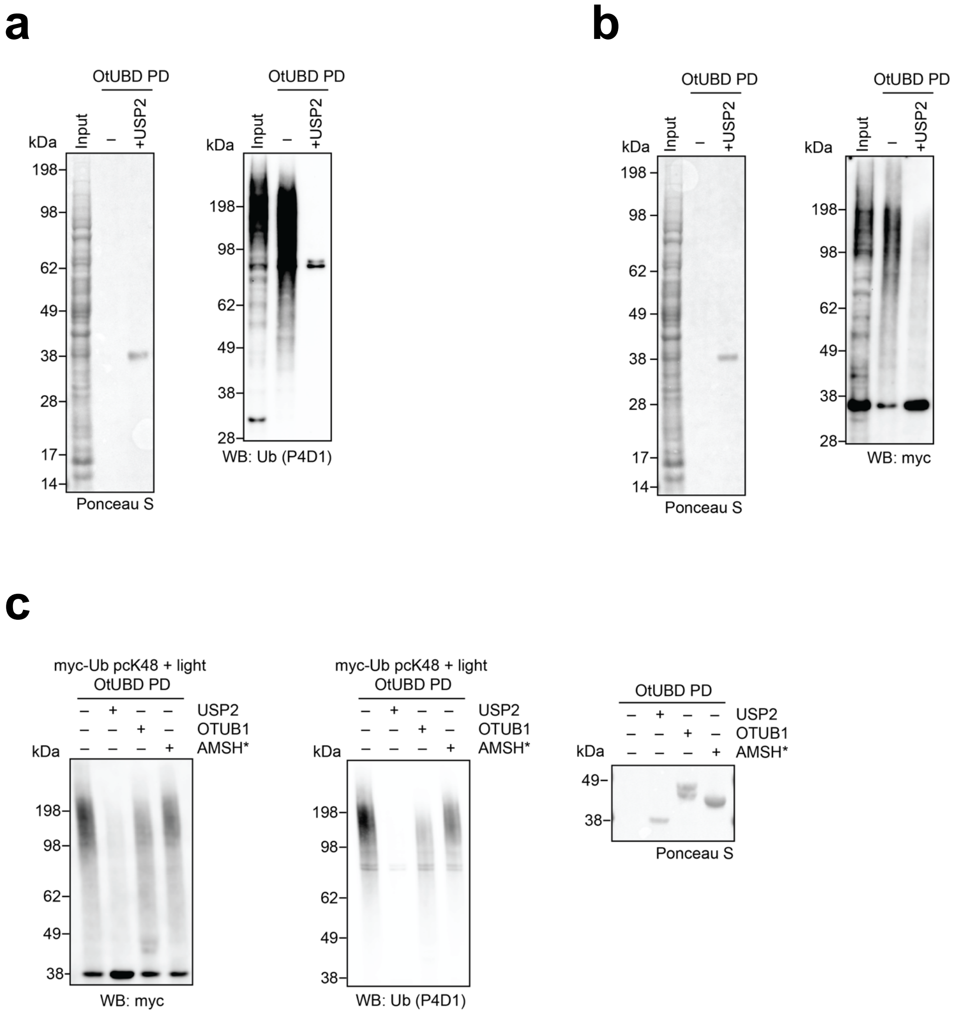

Figure S3. Enrichment and Ubiquitin linkage analysis of Myc-Ub-containing cellular polyubiquitin.

a, b) Enrichment of cellular ubiquitin through pulldowns with biotinylated OtUBD. myc-Ub pcK48 was expressed in HEK293T, decaged with light for 30 min, and lysates were subjected to pulldown and enzyme treatment where indicated (see methods section for details). Samples were analyzed by Ponceau S (whole proteome) and western blotting for ubiquitin and Myc. **c)** Analysis of global ubiquitin linkage types. Cellular polyubiquitin (from samples prepared and enriched as described in panels a-b was treated with the indicated deubiquitinases, comprising the linkage-non-specific DUB USP2 (1 µM), the K48-specific enzyme OTUB1* and the K63-specific enzyme AMSH* (5 µM each).

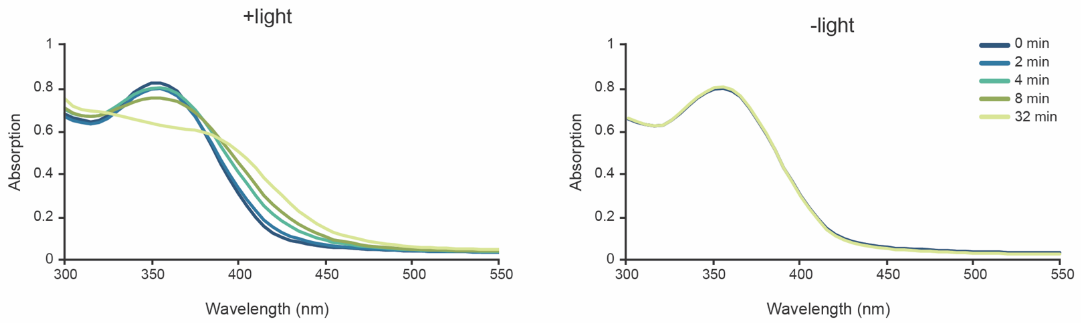

Figure S4. In vitro decaging kinetics of pcK in the experimental setup used for cellular decaging studies.

1 mM of pcK in PBS was irradiated in the exact same culture plate and overall setup as experiments shown in Figures 2-4. Reduction in absorption at ~360 nm indicates occurrence of free caging group. This serves as standardization experiment for reproduction by other laboratories, i.e., for adjusting the irradiation conditions to the ones used in the cellular experiments of the present study. Note the larger time interval between 8 and 32 min, explaining the larger change in absorption.

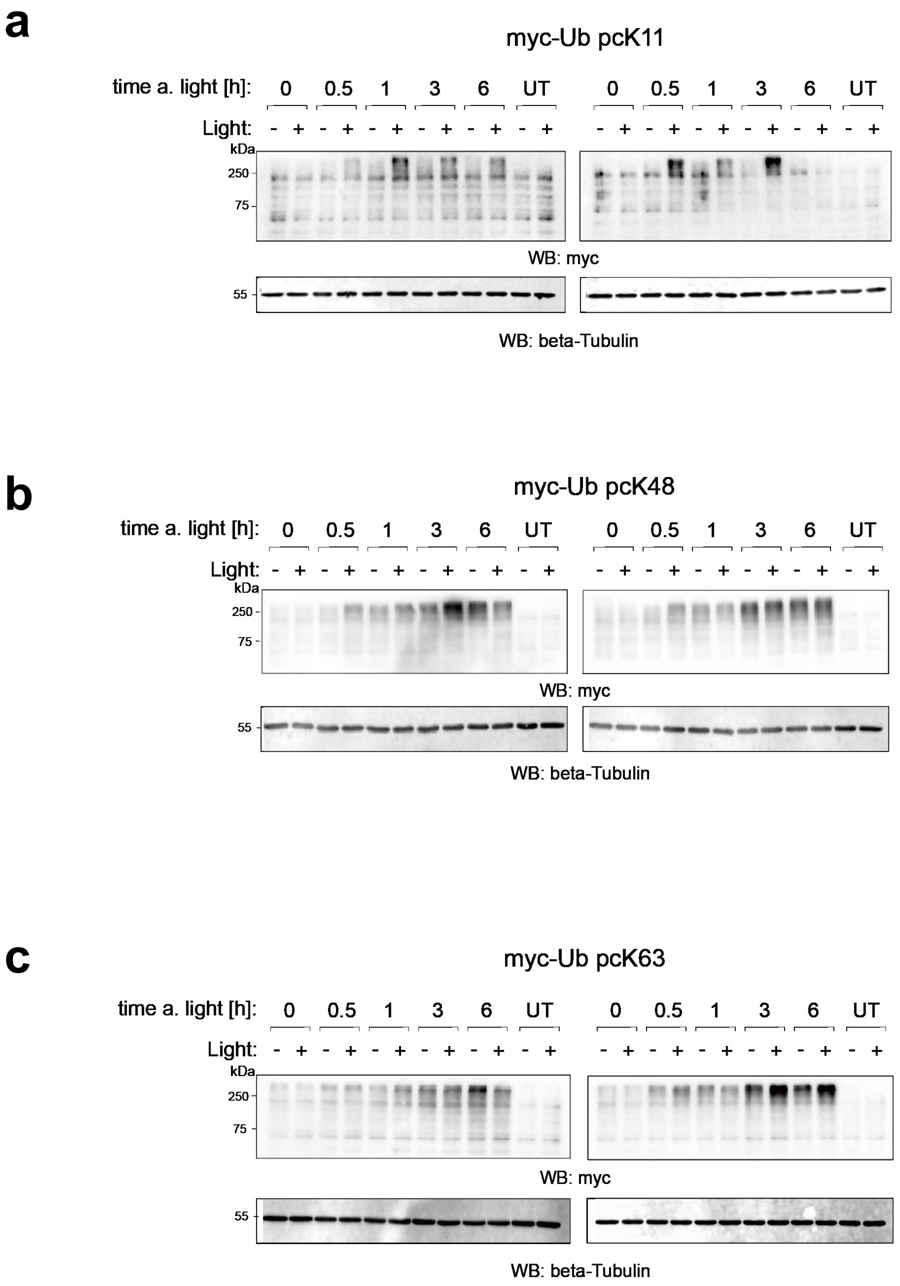

Figure S5. Additional SDS PAGE/anti-myc blots for the analyses of long-term, linkage-specific ubiquitination kinetics after light activation of caged Ub variants.

a) myc-Ub pcK11 **b)** myc-Ub pcK48 **c)** myc-Ub pcK63.

a

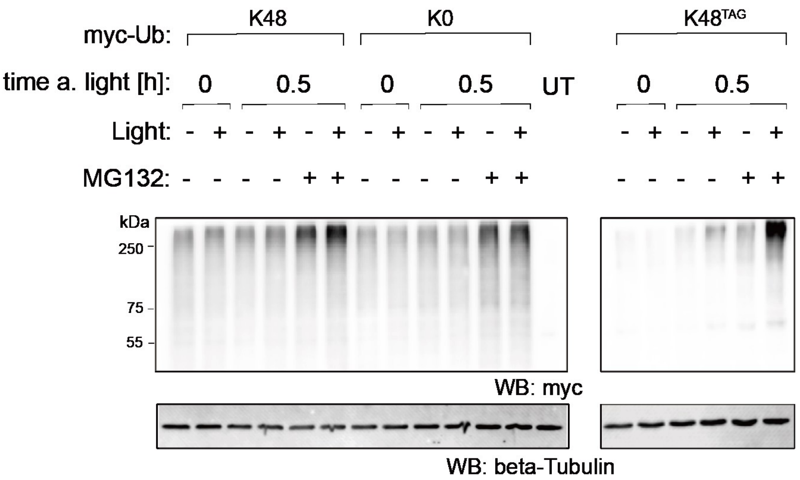

b

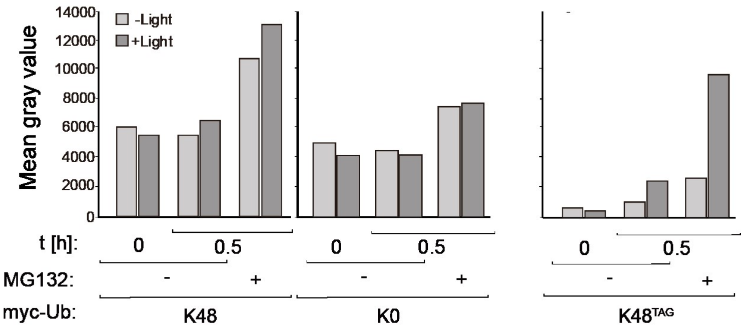

Figure S6. Light alone or MG132 do not have an effect on the increase in high molecular weight myc-Ub in the proteome.

a) HEK293T cells expressing myc-Ub K0, K48 or pcK48 (K48^{TAG} in presence of pcKRS and pcK) for 24 hours were irradiated or not irradiated with light (365 nm, 4 min), treated or not treated with 25 µM MG132, and harvested immediately or after 30 minutes. Only myc-Ub pcK48 showed an increase in the high molecular weight myc-ubiquitinome upon light irradiation, proving that light or MG132 alone are not responsible for this increase. **b)** Bar diagrams showing the mean gray values (arbitrary units) corresponding to the myc-ubiquitinated proteome, normalized to the loading control (beta-Tubulin).

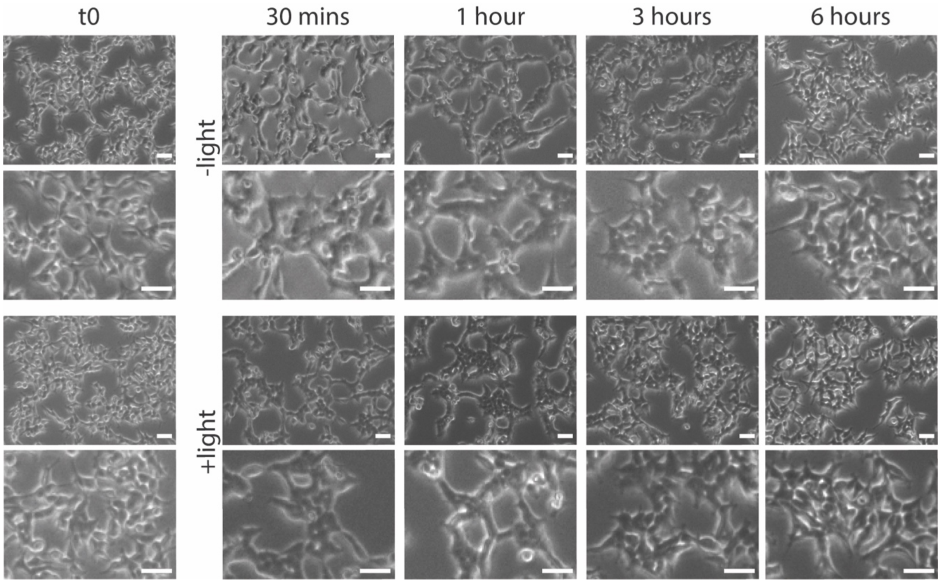

Figure S7. No effect of light on cell morphology.

Light activation under conditions applied in this study indicate no effects on HEK293T cell morphology.

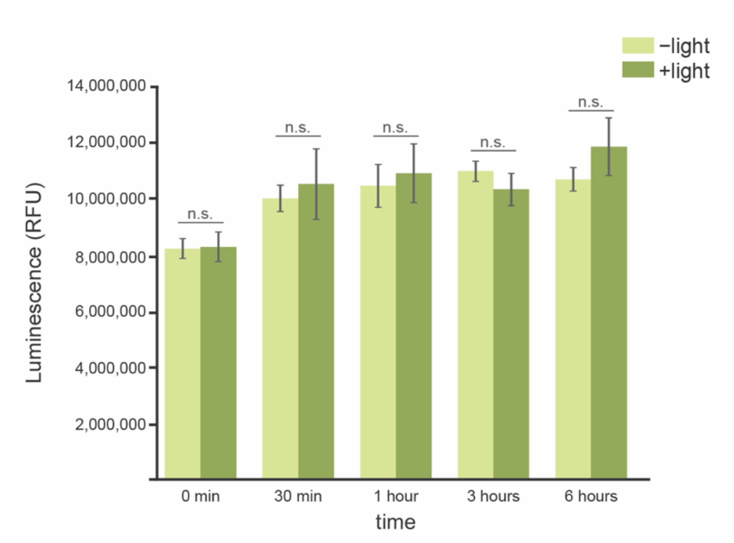

Figure S8. No effect of light on cell viability.

ATP-based luciferase cell viability assay of HEK293T cells before and after light activation (different further cultivation times are indicated) under conditions applied in this study show no effect of light irradiation on cell viability. Error bars are from triplicate experiments and significance has been evaluated via t-test with n.s. (not significant being $p>0.05$).

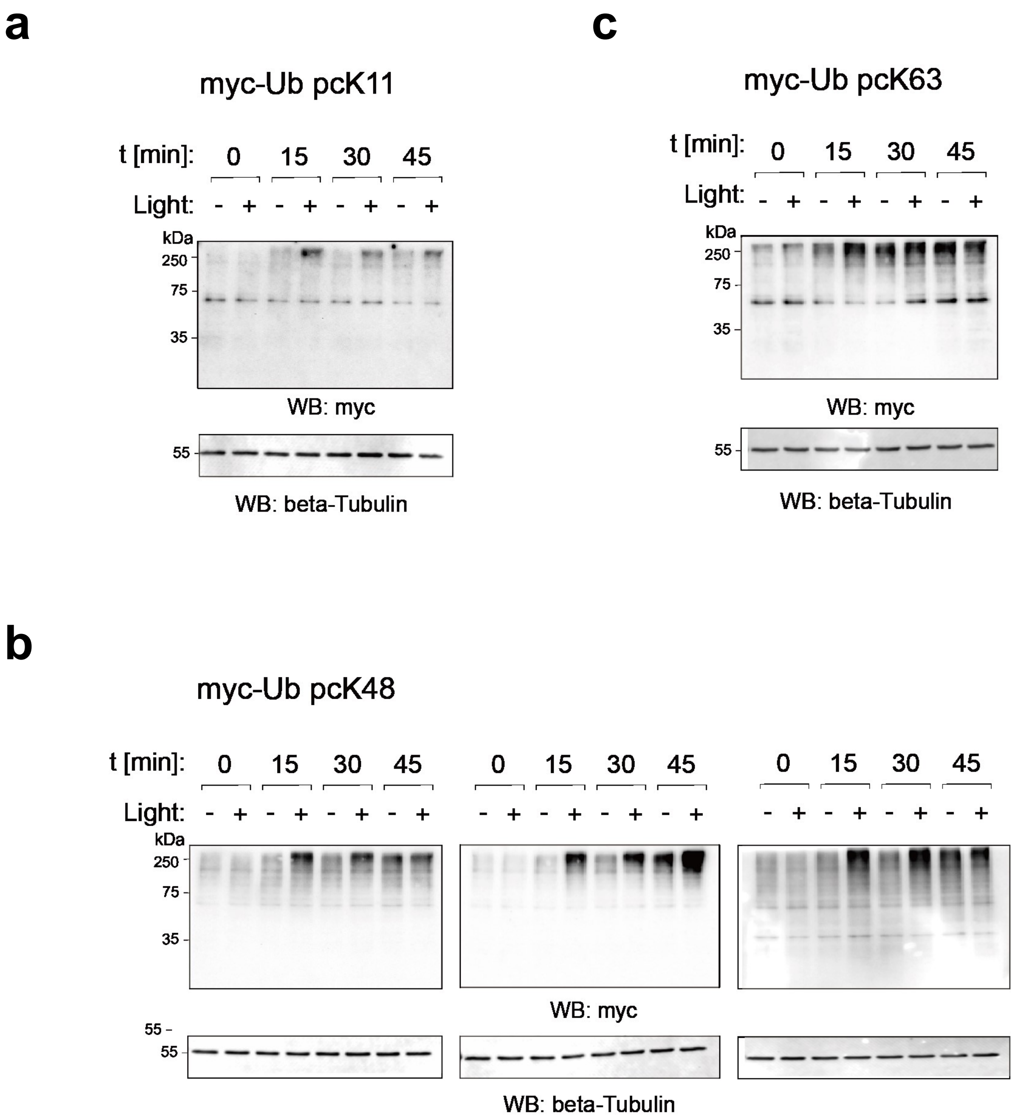

Figure S9. Additional SDS PAGE/anti-myc blots for the analyses of short-term, linkage-specific ubiquitination kinetics after light activation of caged Ub variants.

a) myc-Ub pcK11 **b)** myc-Ub pcK48 **c)** myc-Ub pcK63.

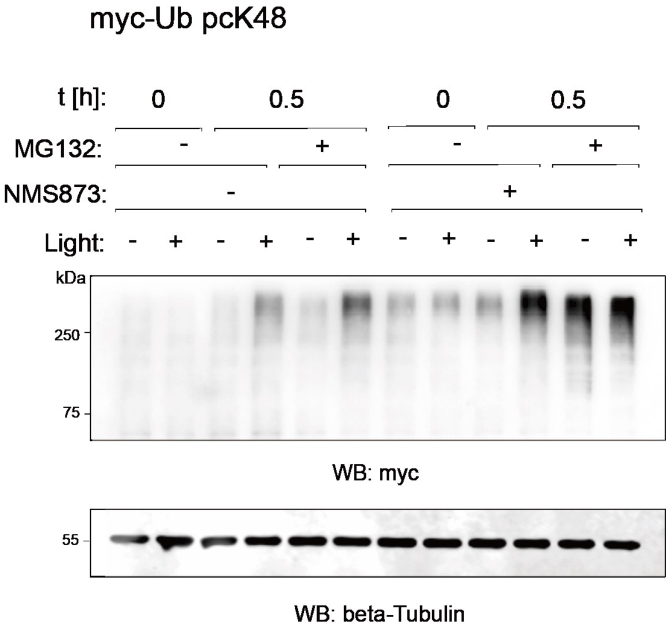

Figure S10. Additional SDS PAGE/anti-myc blot for the analyses of effect of NMS873 on early, K48-specific *de novo* ubiquitome synthesis (t=0.5 h) in presence of MG132.

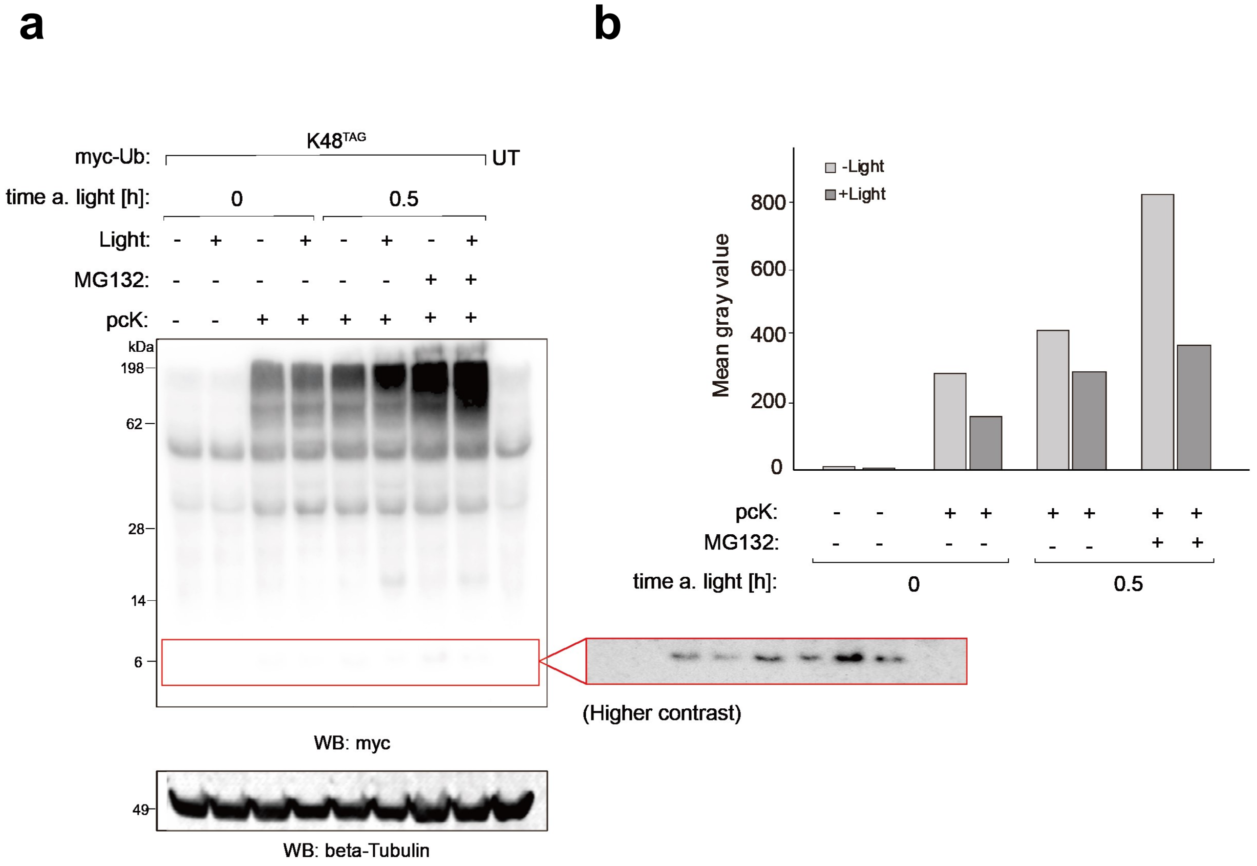

Figure S11. Changes in mono myc-Ub pool in HEK293T cells expressing myc-Ub pcK48 in presence or absence of pcK, with or without light irradiation.

a) HEK293T cells grown in 10 cm plates, co-transfected with myc-Ub pcK48 and pcKRS (a total of 12 µg DNA) and grown in the absence of pcK do not show mono myc-Ub expression. Only when grown in presence of pcK, expression of myc-Ub pcK48 is observed (compare lanes 1-2 with lanes 3-8 in high contrast panel). In order to visualize mono-Ub, samples were run in 4-12% gradient Bis-Tris gels, transferred to 0.2 µm PVDF membranes and stained. **b)** Bar diagrams showing the mean gray values corresponding to the mono myc-ub, that also quantitatively shows changes in this pool upon light irradiation. Values are normalized to the loading control (beta-Tubulin). UT= untransfected cells.

Figure 2b
myc

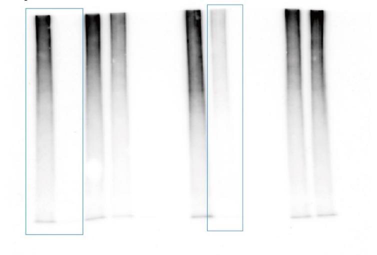

Figure 2c
myc

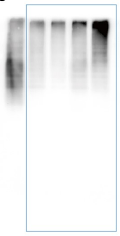

Figure 2e
myc

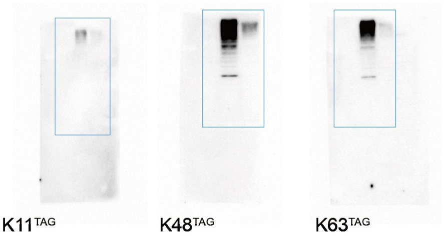

Figure 2f
myc

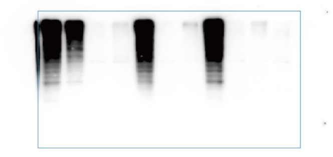

Figure 2g
Ub

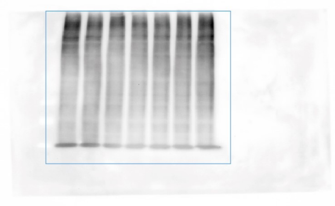

Figure S3a
Ponceau S

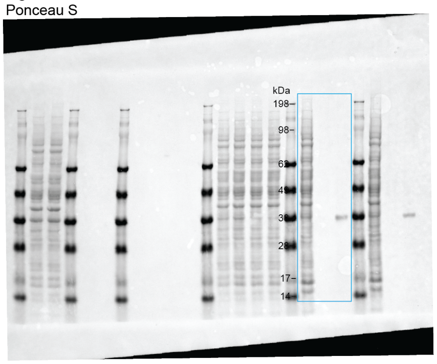

Figure S3a
Ubiquitin

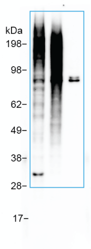

Figure S3b
Ponceau S

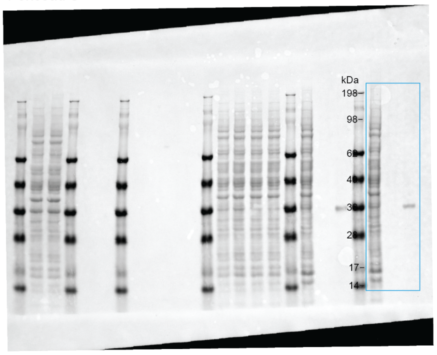

Figure S3b
Myc

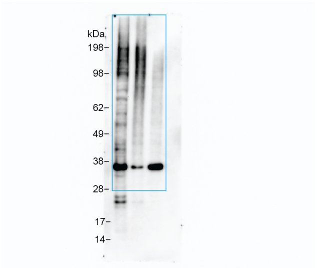

Figure S3c
Myc

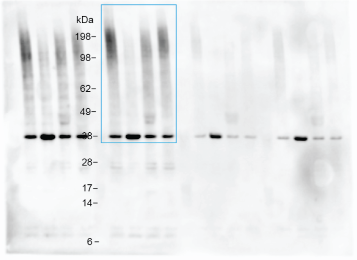

Figure S3c
Ubiquitin

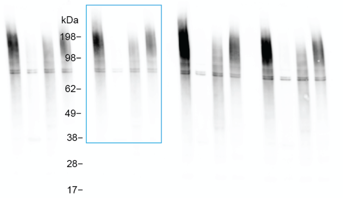

Figure S3c
Ponceau S

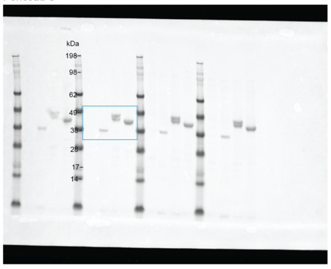

myc- Ub pcK63

Figure 3a
myc

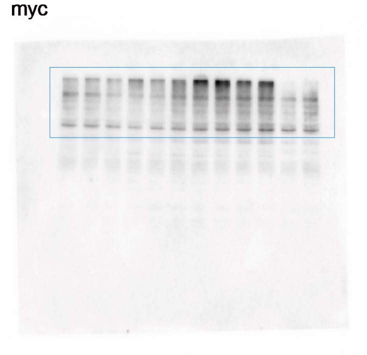

Figure S5c
myc

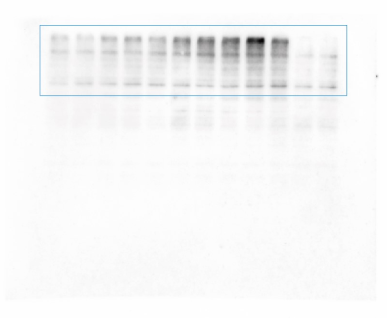

Figure S5c
myc

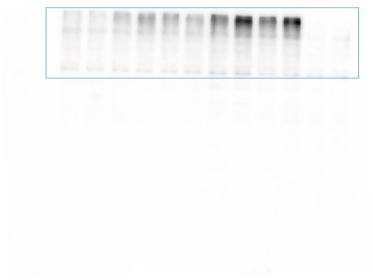

myc- Ub pcK11

Figure S9a
myc

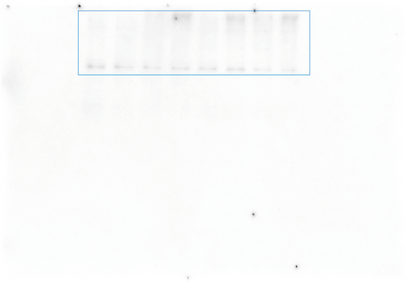

Figure 3b
myc

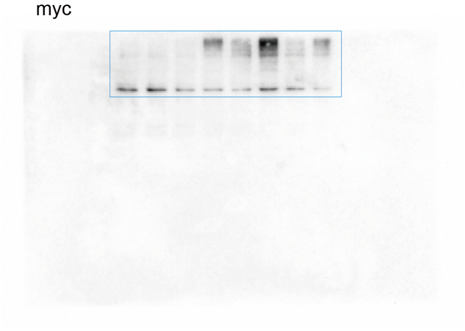

myc- Ub pcK48

Figure S9b
myc

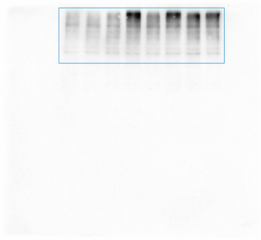

Figure 3b
myc

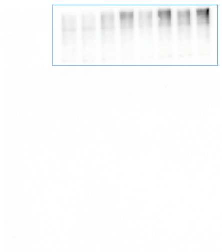

Figure S9b
myc

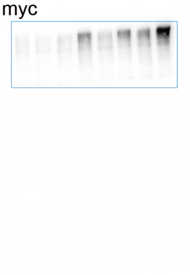

Figure S9b
myc

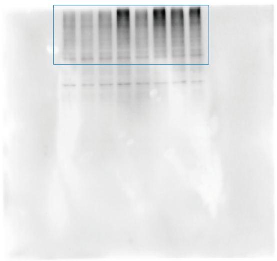

myc- Ub pcK63

Figure 3b
myc

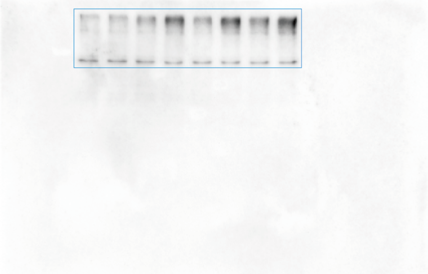

Figure S9c
myc

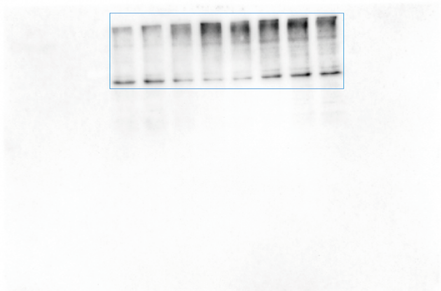

myc- Ub pcK11

Figure 3a
myc

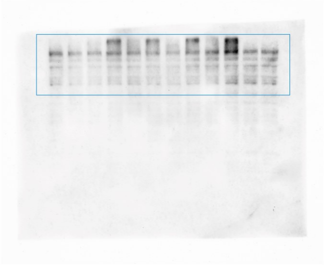

Figure S5a
myc

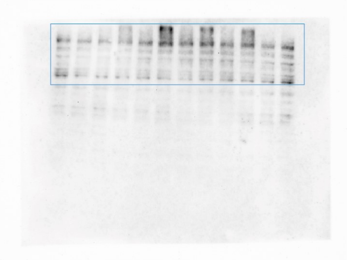

Figure S5a
myc

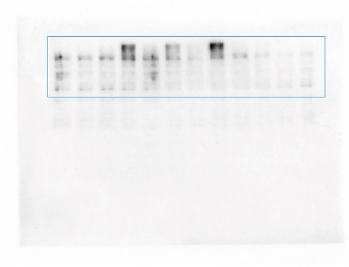

myc- Ub pcK48

Figure S5b
myc

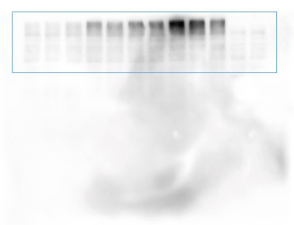

Figure S5b
myc

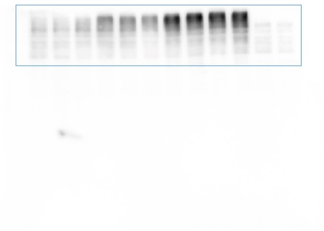

Figure 3a
myc

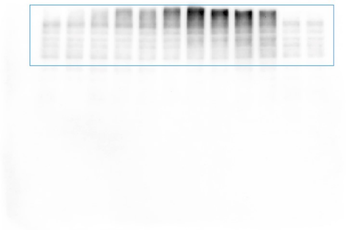

Figure 4c
Myc

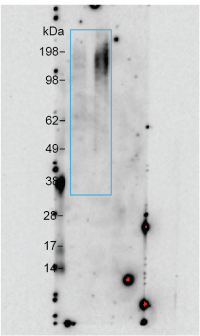

Figure 4b
myc

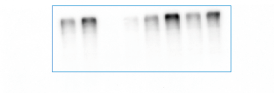

Figure S10
myc

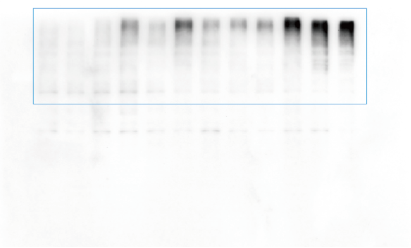

Figure 4d
myc

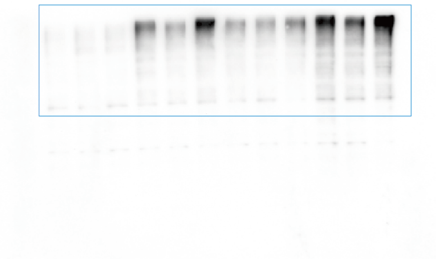

Figure S6
myc

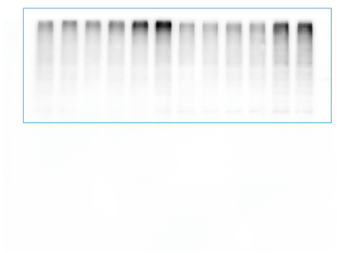

Figure S6
myc

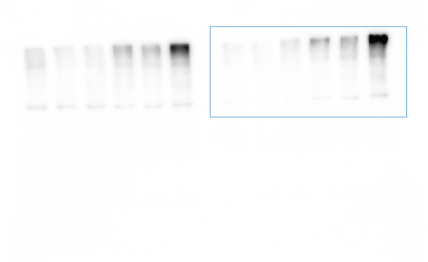

Figure S11
myc

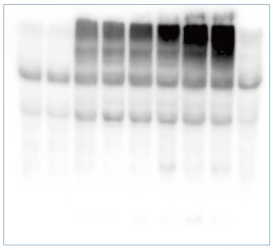

Figure S8. Source data of all immunoblots performed in this work.

MATERIALS AND METHODS

4.1 Construction of Plasmids: Firstly, in order for the incorporation of pcK, a pEVOL[1] based plasmid (p787) with a *M. mazei* PylRS construct, containing the same mutations as reported[2], was constructed. p787 was digested with XbaI and BamHI and ligated into a similarly digested vector backbone obtained from the lab of Prof. Dr. Heinz Neumann, containing four repeats of $tRNA^{Pyl}$ under U6 promoter (pASB654 - SE323_wtPylRS_4xPylT, p2970) to generate the modified PCKRS plasmid (p2946).

A pcDNA3-based Ub plasmid (pcDNA3_HA-Ub, p2972) was constructed by Gibson assembly to insert one copy of Ub with an N-terminal myc-tag (amplified with o4324 and o4327) into plasmid p2932 (amplified with o4325 and o4326) to yield p2945. Quikchange mutagenesis was performed on p2945 to generate the Ub delG76 variant p2950 (using primers o4421 and o4422).

The Ub K0 variant was cloned into the backbone of p2945 using Gibson assembly by first amplifying Ub K0 from p1.9T7minus1 with primers o4948 and o4949 and amplifying p2945 with primers o4946 and o4947, yielding p3041. The Ub K11, K48 and K63 constructs and Ub $K11^{TAG}$, $K48^{TAG}$ and $K63^{TAG}$ constructs were all obtained by performing Quikchanges mutagenesis on p3041. The Ub K11 was generated by Quikchange mutagenesis with o5278 and o5279 to generate p3455, for Ub $K11^{TAG}$, o5280 and o5281 were used to generate p3456, for Ub K48, o5226 and o5227 were used to generate p3439, for Ub $K48^{TAG}$, o5228 and o5229 were used to generate p3440, for Ub K63, o5274 and o5275 were used to generate p3457, and lastly for Ub $K63^{TAG}$, o5276 and o5277 were used to generate p3458.

4.2 Cell culture and transfection: Cells were cultivated in DMEM (Dulbecco's Modified Eagle Medium, w/ 4.5 g/L Glucose, w/o: L-Glutamine, w: Sodium pyruvate, w: 3.7 g/L NaHCO3, PAN Biotech, # P04-03600), supplemented with 10% FBS (South America origin, premium grade, PAN Biotech, # P30-3306), 2 mM L-Glutamine (PAN Biotech, # P04-80100), 100 U/mL Penicillin and 0.1 mg/mL Streptomycin (PAN Biotech, # P06-07100) in a sterile, humidified incubator (≥95%) at 37°C and a CO_2 level of 5 %.

For transfection, cells were seeded in 6-well cell culture plates (Sarstedt), a day before so that they reached 70-80% confluency at the time of transfection. Transient plasmid transfection was performed using Lipofectamine-2000 (Thermo Fisher Scientific, # 11668019) following the supplier’s guidelines. For transfection, 1 µg of DNA was used in case of non-amber containing constructs and a total of 2 µg of DNA was used in case of the amber containing constructs (except for experiment in Fig. S4 where cells were cultivated in 10 cm dishes and a total of 12 µg DNA was used for transfection).

4.3 Synthesis of pcK: The ncAA photocaged lysine (pcK) was synthesized according to Gautier *et al.* 2010[2].
^{1}H NMR: (400 MHz, D_2O): δ 7.49 (s, 1H, Ar-H), 7.13 (s, 1H, Ar-H), 6.08-6.18 (m, 3H, CH_2, CH), 4.00-4.14 (m, 1H, CH) 3.30-2.98 (m, 3H, NH, CH_2), 1.85-2.12 (m, 2H, CH_2), 1.32-1.67 (m, 7H, 2x CH_2, CH_3).
^{13}C NMR: (100 MHz, D_2O): δ 172.17, 157.29, 152.66, 146.91, 140.65, 135.91, 105.40, 104.78, 103.40, 69.19, 52.77, 39.73, 29.35, 28.31, 21.43, 20.84.
HRMS (ESI): Observed m/z 382.1261 $(M - H)^-$, Calculated m/z for $[C_{16}H_{20}N_3O_8]^-$ = 382.1256.

4.4 Light activation: HEK293T cells were grown in 6-well cell culture plates (Sarstedt, cells for DUB assays were cultivated in 10 cm dishes) transfected with plasmids containing amber Ub variants (Ub K11^{TAG}, K48^{TAG} and K63^{TAG}) and p2946 (modified PCKRS) (Figure S1. Data from Fig. S4 are from cultivation in 10 cm dishes) as described under 4.2. After adding the transfection mixture, 0.32 mM of pcK (synthesized as describe above or purchased from Sigma Aldrich, # 915793) was added directly in the growth media and the cells were allowed to express the protein for 24 hours. For treatment with light[3], the growth medium containing pcK was exchanged with warm DPBS (Dulbecco's Phosphate-Buffered Saline, Mg/Ca free, PAN Biotech, #P04-36500) and placed on a 365 nm UV-transilluminator (Witeg DH. WUV00010, 6x 15 W) for 4 minutes (decaging of this and related noncanonical amino acids is also possible with 405 nm light and higher temporal resolution using suited higher energy light sources such as lasers or LEDs). Immediately after the light treatment, the DPBS was exchanged and pre-warmed medium (without pcK, but with 25 µM MG132; Enzo Life Sciences, # BML-PI102) was added and the cells were further grown in a humidified incubator (≥95%) at 37ºC and a CO_2 level of 5 %.). Cells were harvested at indicated time points. For in vitro irradiation experiments of Fig. S9, 1 mM of pcK was dissolved in PBS and irradiated under identical conditions as used in cell experiments for different times. Samples were taken at indicated time points and analyzed by UV absorption measurements (Perkin Elmer Envision Multimode Plate Reader 2104). Cell viability assays were conducted with cells irradiated for 4 min under identical conditions as used in other cell experiments after different additional cultivation times using the Cell-Titer Glow assay (Promega) according to the manufactures conditions.

4.5 Cell lysis and Immunoblotting: Cells were trypsinised and collected in microcentrifuge tubes (Sarstedt). The cell pellets were lysed with 200 µL of lysis buffer (0.5% IGEPAL, 50 mM Tris (pH=7.5), 150 mM NaCl, 10% glycerol) containing protease inhibitor (Roche complete™, 11697498001), 0.1 mM PMSF and in addition, 20 mM NEM (Thermo Fisher Scientific, 23030) and 2 mM EDTA for inhibiting DUB activity. After completely dissolving the pellets with lysis buffer, the samples were incubated at 4ºC for 60 min, shaking at 700 rpm. Crude lysates were cleared by centrifugation at full speed at 4ºC for 15 min. The lysates were collected and protein concentrations were determined using Bicinchoninic Acid (BCA) protein assay. Samples were prepared using 4X Laemmli sample buffer and loaded on 12% SDS gels.

Proteins were transferred to 0.2 µm polyvinylidene fluoride (PVDF) membranes using a Trans-Blot Turbo system (1.9 A, 25 V, 7 min; Bio-Rad, # 1704150) and transfer kit (Bio-Rad, # 1704272). The membranes were blocked with 5% (m/v) nonfat milk in TBS-T (TBS + 0.1% v/v Tween-20) buffer and incubated with indicated primary antibodies (rabbit monoclonal anti-myc 71D10, 1:1000 dilution, Cell Signaling, # 2278; rabbit monoclonal anti-ubiquitin E412J, 1:1000 dilution, Cell Signaling, # 43124; mouse anti-β-Tubulin, 1:2000 dilution, Cell Signaling, # 86298) overnight at 4°C with gentle shaking. The membranes were then incubated with the respective secondary antibody (HRP linked rabbit IgG, 1:5000, Merck, # GENA934; Dylight680-conjugated goat anti-mouse secondary antibody, 1:10000 dilution, Invitrogen, # 10797775). The chemiluminescent reaction was initiated using a Clarity Western ECL substrate and enhancer (Bio-Rad # 1705061; # 1705062) and chemiluminescent images were taken on a Bio-Rad ChemiDoc™ imaging system. Fluorescent images of the blots

were taken on the Odyssey® DLx imaging system (LI-COR). Typically, the chemiluminescent staining and imaging were done first, followed by the fluorescent staining and imaging of the β-Tubulin loading control. The myc or Ub smears from the individual images of the immunoblots and the β-Tubulin bands were quantified using Fiji Image J software[4,5].

For ubiquitome enrichment and enrichment of branched ubiquitin-chains, proteins were transferred to 0.2 µm nitrocellulose membranes using a Trans-Blot Turbo system (1.0 A, 25 V, 30 min; Bio-Rad, # 1704150) with a transfer kit (Bio-Rad, # 1704271). Membranes were blocked with 5% (w/v) nonfat milk in PBS-T (PBS + 0.1% v/v Tween-20), and immunoblotting was carried out as described above with the following antibodies: mouse monoclonal anti-myc 9E10, 1:250 dilution, Santa Cruz Biotechnology, # 9E10 in PBS-T; mouse monoclonal anti-ubiquitin P4D1, 1:1000 dilution, Cell Signaling, # 3936 in PBS-T; HRP-linked mouse IgG, 1:5000, Merck, # GENXA931.

4.6 Immunostaining and Flow Cytometry: For analysis of expression of the different non-amber Ub variants, HEK293T cells expressing the respective constructs were harvested 24 hours after transfection (adding 10 µM MG132 5 hours before harvesting). Cells were washed with DPBS, trypsinized with Trypsin 0.05% / EDTA 0.02% (PAN Biotech, # P10-038100) for 5 minutes at 37ºC and blocked with full DMEM medium. Harvested cells were washed once with DPBS and fixed with medium A (Fix & Perm kit, Thermo Fisher Scientific, # GAS004) for 15 min at RT followed by washing with wash buffer (PBS + 5% FBS + 0.1% NaN_3). Then the fixed cells were permeabilized with medium B for 20 min. Thereafter, the cells were blocked with blocking buffer (PBS+1% BSA + 0.05% Tween) overnight at 4°C with gentle shaking. For immunostaining, mouse monoclonal anti-myc (9E10) (Santa Cruz Biotechnology, # sc-40) primary antibody was added to the cells in 1:500 dilution followed by incubation at RT for 1 h. After washing with PBST buffer, Alexa Fluor 488 conjugated goat anti mouse (Thermo Scientific, # A-11001) secondary antibody was incubated in 1:1000 dilutions for 1 h. Cells were then washed with PBST and PBS buffer before being measured by Flow Cytometry (Sony, # LE-SH800SFP). FCM results were analysed using Cell Sorter Software (Sony). Untransfected cells (UT) were used as a control.

4.7 UbiCRest assays: HEK293T cells were grown in 10 cm dishes and light activated as described above and were pelleted after indicated time, washed with ice-cold PBS and stored at -80 °C until further processed. The cell pellet was lysed in urea lysis buffer (4 M urea, 50 mM Tris HCl pH 8, 150 mM NaCl, 1% IGEPAL, 2 mM EDTA, 5% glycerol, 1x EDTA-free protease inhibitor cocktail, 1 mM PMSF, 10 µM PR619, 20 mM *N*-ethylmaleimide, 4 mM 1,10-phenanthroline) before being homogenised using sonication for 10 s (2 s on and 2 s off) at 10% amplitude and cleared by centrifuged for 10 min at 14,000 x g and 4 °C.

Biotinylated OtUBD was prepared as described elsewhere[6] and 4 nmol of the biotinylated ubiquitin-binding entities were immobilized on 67 µL high-capacity neutravidin agarose bead slurry (Pierce, Thermo Fisher Scientific) for 1 h at 4 °C with rotation. Excess reagent was washed away with ice-cold PBS. Beads were then equilibrated in lysis buffer.

Protein concentrations between the conditions was adjusted per Bradford assay and 1 mg of protein suspension were added to the pre-immobilized pull-down reagent and incubated for 2 h at 4 °C with rotation. Beads were

pelleted by centrifugation at 500 x g for 1 min and the supernatant was removed. The resin was washed once with diluted lysis buffer, once with high-salt buffer (50 mM Tris HCl pH 8, 1 M NaCl), once with ice-cold PBS and twice with ice-cold water. Subsequently, ubiquitinated proteins were eluted from OtUBD using 35 µL of a 100 mM glycine solution at pH 2.5 with an incubation time of 5 min at room temperature. Beads were pelleted by centrifugation at 500 x g for 1 min. The supernatant was transferred to a new tube and brought immediately to a neutral pH through addition of 3.5 µL of 1 M Tris at pH 9.0. This step was repeated once, the resulting supernatants were combined and supplemented with final concentrations of 5 mM DTT, 5 mM $MgCl_2$ and 100 mM NaCl.

For UbiCRest assays[7,8], supernatants were incubated with USP2 (1 µM), OTUB1* (5 µM), AMSH* (5 µM) for 90 min at 37 °C. The reactions were stopped by adding 4 x LDS sample buffer (supplemented with 50 mM DTT) and visualized by SDS-PAGE and Western blotting.

4.8 Pulldown with NbSL3.3Q nanobody-coupled agarose beads and cell lysate: Cells from one 10 cm dish (treated as in 4.7) per condition were pelleted, washed with ice-cold PBS and stored at -80 °C until further processed. The cell pellet was lysed in immunoprecipitation buffer (50 mM Tris HCl pH 8, 150 mM NaCl, 2 mM EDTA, 1 mM PMSF, 1% IGEPAL, 20 mM *N*-ethylmaleimide, 1x EDTA-free protease inhibitor cocktail) before being homogenized using sonication for 10 s (1 s on and 1 s off) at 10% amplitude and cleared by centrifuged for 10 min at 14,000 x g and 4 °C.

40 µL of NbSL3.3Q[9] nanobody-coupled agarose bead slurry per condition was pre-washed with ice-cold PBS and was equilibrated in immunoprecipitation buffer. 1 mg of cell lysate was added to the beads and incubated for 1 h at 4 °C with rotation. The beads were washed four times with immunoprecipitation buffer (containing 300 mM NaCl) and proteins were eluted with 2x LDS sample buffer (supplemented with 50 mM DTT) with boiling for 5 min at 95 °C. Beads and the enriched branched ubiquitin chains were separated by pelleting of the beads at 500 x g for 1 min. Samples were analysed by SDS-PAGE and Western blotting as described above.

SUPPLEMENTARY TABLES

Table S1. Oligonucleotides for plasmids construction.

Name	Sequence (5'→3')
o4324_ShP	CTGATCTCAGAGGAGGACCTGAGCCTGTCTAGAGGCATG
o4325_ShP	CCTCTGAGATCAGCTTCTGCTCGCCCATGGTGGCTAGC
o4326_ShP	GGTGGGATGACTAGCTGAATCGGTAGGAATTC
o4327_ShP	GAATTCCTACCGATTCAGCTAGTCATCCCACC
o4421_ShP	CCGTCTCAGAGGTATGACTAGCTGAATC
o4422_ShP	GATTCAGCTAGTCATACCTCTGAGACGG
o4946_SuB	GCCTCTAGACAGGCTCAGGTC
o4947_SuB	ATCGGTAGGAATTCGCGGCCG
o4948_SuB	GAGGAGGACCTGAGCCTGTCTAGAGGCATGCAGATCTTCGTGAGGACC
o4949_SuB	CGGCCGCGAATTCCTACCGATTCACCCACCTCTGAGACGGAG
o5226_SuB	GATCTTTGCTGGGAAACAGCTGGAAG
o5227_SuB	CTTCCAGCTGTTTCCCAGCAAAGATC
o5228_SuB	GATCTTTGCTGGGTAGCAGCTGGAAG
o5229_SuB	CTTCCAGCTGCTACCCAGCAAAGATC
o5274_SuB	GACTACAACATCCAGAAAGAGTCCACCCTG
o5275_SuB	CAGGGTGGACTCTTTCTGGATGTTGTAGTC
o5276_SuB	GACTACAACATCCAGTAGGAGTCCACCCTG
o5277_SuB	CAGGGTGGACTCCTACTGGATGTTGTAGTC
o5278_SuB	GGACCCTGACTGGTAAGACCATCACTCTCG
o5279_SuB	CGAGAGTGATGGTCTTACCAGTCAGGGTCC
o5280_SuB	GGACCCTGACTGGTTAGACCATCACTCTCG
o5281_SuB	CGAGAGTGATGGTCTAACCAGTCAGGGTCC

Table S2. Protein coding sequences used in this study.

Protein	Sequence (Myc-Ub)
Ub wt	EQKLISEEDLSLSRGMQIFVKTLTGKTITLEVEPSDTIENVKAKIQDKEGIPPDQQRLIFAGKQLEDGRTLSDYNIQKESTLHLVLRLRGGMTS
Ub nc	EQKLISEEDLSLSRGMQIFVKTLTGKTITLEVEPSDTIENVKAKIQDKEGIPPDQQRLIFAGKQLEDGRTLSDYNIQKESTLHLVLRLRG-MTS
Ub K0	EQKLISEEDLSLSRGMQIFVRTLTGRTITLEVEPSDTIENVRARIQDREGIPPDQQRLIFAGRQLEDGRTLSDYNIQRESTLHLVLRLRGG
Ub K11	EQKLISEEDLSLSRGMQIFVRTLTGKTITLEVEPSDTIENVRARIQDREGIPPDQQRLIFAGRQLEDGRTLSDYNIQRESTLHLVLRLRGG
Ub pcK11	EQKLISEEDLSLSRGMQIFVRTLTGpcKTITLEVEPSDTIENVRARIQDREGIPPDQQRLIFAGRQLEDGRTLSDYNIQRESTLHLVLRLRGG
Ub K48	EQKLISEEDLSLSRGMQIFVRTLTGRTITLEVEPSDTIENVRARIQDREGIPPDQQRLIFAGKQLEDGRTLSDYNIQRESTLHLVLRLRGG
Ub pcK48	EQKLISEEDLSLSRGMQIFVRTLTGRTITLEVEPSDTIENVRARIQDREGIPPDQQRLIFAGpcKQLEDGRTLSDYNIQRESTLHLVLRLRGG
Ub K63	EQKLISEEDLSLSRGMQIFVRTLTGRTITLEVEPSDTIENVRARIQDREGIPPDQQRLIFAGRQLEDGRTLSDYNIQKESTLHLVLRLRGG
Ub pcK63	EQKLISEEDLSLSRGMQIFVRTLTGRTITLEVEPSDTIENVRARIQDREGIPPDQQRLIFAGRQLEDGRTLSDYNIQpcKESTLHLVLRLRGG

SUPPLEMENTARY REFERENCES

[1] T. S. Young, I. Ahmad, J. A. Yin, P. G. Schultz, An enhanced system for unnatural amino acid mutagenesis in E. coli. *J Mol Biol* **2010**, *395*, 361-374.

[2] A. Gautier, D. P. Nguyen, H. Lusic, W. An, A. Deiters, J. W. Chin, Genetically encoded photocontrol of protein localization in mammalian cells. *J Am Chem Soc* **2010**, *132*, 4086-4088.

[3] S. Palei, B. Buchmuller, J. Wolffgramm, A. Munoz-Lopez, S. Jung, P. Czodrowski, D. Summerer, Light-Activatable TET-Dioxygenases Reveal Dynamics of 5-Methylcytosine Oxidation and Transcriptome Reorganization. *J Am Chem Soc* **2020**, *142*, 7289-7294.

[4] C. T. Rueden, J. Schindelin, M. C. Hiner, B. E. DeZonia, A. E. Walter, E. T. Arena, K. W. Eliceiri, ImageJ2: ImageJ for the next generation of scientific image data. *BMC Bioinformatics* **2017**, *18*, 529.

[5] C. A. Schneider, W. S. Rasband, K. W. Eliceiri, NIH Image to ImageJ: 25 years of image analysis. *Nat Methods* **2012**, *9*, 671-675.

[6] K. Wendrich, K. Gallant, S. Recknagel, S. Petroulia, N. H. Kazi, J. A. Hane, S. Führer, K. Bezstarosti, R. O'Dea, J. Demmers, M. Gersch, Discovery and mechanism of K63-linkage-directed deubiquitinase activity in USP53. *Nat Chem Biol* **2024**, *in press*.

[7] T. E. Mevissen, M. K. Hospenthal, P. P. Geurink, P. R. Elliott, M. Akutsu, N. Arnaudo, R. Ekkebus, Y. Kulathu, T. Wauer, F. El Oualid, S. M. Freund, H. Ovaa, D. Komander, OTU deubiquitinases reveal mechanisms of linkage specificity and enable ubiquitin chain restriction analysis. *Cell* **2013**, *154*, 169-184.

[8] M. A. Michel, P. R. Elliott, K. N. Swatek, M. Simicek, J. N. Pruneda, J. L. Wagstaff, S. M. Freund, D. Komander, Assembly and specific recognition of k29- and k33-linked polyubiquitin. *Mol Cell* **2015**, *58*, 95-109.

[9] S. M. Lange, M. R. McFarland, F. Lamoliatte, T. Carroll, L. Krshnan, A. Perez-Rafols, D. Kwasna, L. Shen, I. Wallace, I. Cole, L. A. Armstrong, A. Knebel, C. Johnson, V. De Cesare, Y. Kulathu, VCP/p97-associated proteins are binders and debranching enzymes of K48-K63-branched ubiquitin chains. *Nat Struct Mol Biol* **2024**, *in press*.
